# Bayesian latent class models to determine diagnostic sensitivities and specificities of two point of care rapid tests (Selma plus, Dipslide) for the detection of *Streptococcus uberis* associated with mastitis in dairy cows

**DOI:** 10.3389/fvets.2022.1062056

**Published:** 2022-12-13

**Authors:** David Rediger, Marc André Butty, Sonja Kittl, Michèle Bodmer, Sonja Hartnack

**Affiliations:** ^1^Clinic for Ruminants, Vetsuisse Faculty, University of Bern, Bern, Switzerland; ^2^Institute for Veterinary Bacteriology, Vetsuisse Faculty, University of Bern, Bern, Switzerland; ^3^Section of Veterinary Epidemiology, Vetsuisse Faculty, University of Zurich, Zurich, Switzerland

**Keywords:** bovine mastitis, point-of-care diagnostic, rapid culture test, bacteriological culturing, Bayesian latent class analysis, agreement

## Abstract

**Introduction:**

Development and validations of accurate mastitis diagnostics are crucial to make timely and evidence-based decisions on mastitis therapy in order to reduce its impact on productivity, animal welfare and practicing the prudent use of antimicrobials on dairy farms.

**Methods:**

The objectives of this study were to assess the agreement between test results from reference laboratory and two point of care tests (Selma plus, Dipslide) and to estimate the test accuracies with Bayesian latent class models (BLCMs). In total of 509 single quarter milk samples from cows with mastitis were included in the study.

**Results:**

Among all analyzed mastitis pathogens, *Streptococcus* spp. was detected in up to one third of all analyzed samples and for Selma all Streptococcus samples were considered as *Streptococcus uberis*. The agreement (κ) when comparing two tests varied greatly depending on the bacteria, ranging from no agreement to good agreement (κ = negative to 0.86) depending on the prevalence of identified pathogens. Based on BLCMs to assess diagnostic test accuracies for the pathogen *Streptococcus uberis*, posterior sensitivities of 76, 71, and 64% for Selma plus, Dipslide and laboratory standard culture and specificities of 93%, 98% for Selma and Dipslide, respectively, were obtained.

**Discussion:**

The two point of care rapid culture systems Dipslide and Selma plus plate can provide important preliminary pathogen identification for targeted mastitis therapy, especially when general information about growth and a rough classification of the bacteria into groups have an impact on treatment strategy. The two evaluated rapid culture systems, Dipslide and Selma plus plate, show good test accuracies for *Streptococcus uberis* at least at genus level. Therefore, using these tests may contribute to prudent use of antibiotics.

## Introduction

Globally, bovine mastitis is a major issue among ruminant infectious diseases, impacting dairy farming in terms of animal welfare, economical losses (reduced milk yield and quality, treatment costs and involuntary culling costs) and antimicrobial usage (1, 2). Intramammary infection (IMI) is commonly caused by microorganisms. Treatment and prevention of mastitis have been recognized as the most common reason for antibiotic use in adult dairy cattle (3–5). Based on clinical judgement, the therapeutic use of antibiotics is possibly indicated for mastitis cases and justified on animal welfare grounds. However, the large-scale use of antibiotics in dairy farming is viewed critically due to the development and spread of antimicrobial resistance. Furthermore, residues must not be present in milk and meat, as they can endanger human health. Additionally, there is an economic loss for the producer due to non-saleable milk during the period of treatment and the following milk withholding period (1, 2, 6, 7).

In consequence, developing and validating new diagnostic tools and evidence-based strategies for mastitis control are crucial. This is important not only to keep production losses as low as possible, but also to implement the guidelines of prudent use of antimicrobials in dairy farming. Roberson et al. estimated that antibiotics labeled for intramammary use would not be justified in 50–80% of clinical mastitis cases, mainly due to no growth, growth of gram-negative bacteria or yeast in the milk culture (8). Therefore, knowing the causative pathogen supports decision-making concerning antimicrobial treatment in cases of clinical mastitis. Time and accuracy of detection methods are essential prerequisites to enable targeted treatment with or without antimicrobials. To determine the etiology of mastitis will not just influence the decision about the need for antibiotic treatment but will also give information about optimal duration of the treatment, as treatment duration recommendations will vary among pathogens (9).

Besides clinical mastitis, concerns about management of subclinical mastitis are growing. Several European studies show that the group of minor pathogens - known to cause predominantly subclinical mastitis - has become the most common bacterial group isolated from quarter milk samples (10–13). Economic consequences of subclinical mastitis are considered to be equal to clinical mastitis and are accounting for up to two thirds of the economic loss (14–16). Subclinical mastitis can only be detected by the measurement of inflammatory components and pathogens in the milk (17). Somatic Cell Count (SCC) has been shown to be an excellent predictor for subclinical mastitis and reduced milk yield (18). Besides production loss, increased bulk milk tank SCC and the risk of excreting microorganisms that might infect other cows, are important considerations when talking about identifying cows having a longer period of chronically elevated SCC (17). In Switzerland, milk quality is of major interest, as about 40% of the milk production is processed to cheese (mainly raw milk cheese) (19). Subclinical mastitis changes the composition of milk, and these changes may affect milk processing (20). Furthermore, the penalty threshold of bulk milk SCC in Switzerland is set at 350'000 SCC/ml (geometric mean of 4 measures within 5 months) by law (21). Most dairy producers receive bonus payments for maintaining a low bulk tank somatic cell count (< 100'000 SCC/ml). All these reasons are motivating Swiss dairy farmers to have effective control of subclinical mastitis.

On the other hand, antimicrobial usage in veterinary medicine in Switzerland is under scrutiny and a national program for reduction of antimicrobial resistance was launched by the government in 2016 (22). In the framework of the national program a federal database for recording of antimicrobial treatments in domestic animals is in place since the beginning of 2019. All antibiotics administered to domestic animals are recorded at animal age group level by the prescribing veterinarian. In the context of reduced income by selling and administering antimicrobial drugs, the increased implementation of diagnostics not only contributes to prudent use of antibiotics, but also provides another source of income for veterinarians.

Summarized, bacteriological results from subclinical and clinical mastitis on individual cow level will allow establishing control measures and targeted mastitis control on herd level. Identification of mastitis causing pathogens on individual farms is a key component of managing mastitis and mastitis treatment (23).

Isolation of a bacterial pathogen from milk of an infected quarter is considered the gold or reference standard for diagnosis of IMI (24). Pathogens involved in mastitis are usually identified by standard bacteriological laboratory culture, where diagnostic specificity is usually very high- depending on the species close to 100%- but diagnostic sensitivity can be significantly lower (25). Additional species identification of cultured bacteria by MALDI-TOF mass spectrometry is a powerful and reliable technique to identify mastitis causing pathogens (26). Besides bacteriological culturing, PCR approaches have been established for the diagnosis of mastitis pathogens (27, 28). However, a diagnostic test with perfect accuracy does not exist. Thus, when evaluating the diagnostic test accuracies - sensitivity and specificity- of a new test by considering bacteriological culture as a gold standard, the resulting estimated sensitivities and specificities might be biased. In the absence of a gold standard, Bayesian latent class models (BLCM) are a valid approach for evaluating diagnostic test accuracies (25, 27).

In addition, all of the above-mentioned diagnostic techniques require substantial laboratory infrastructure and cannot be performed outside of professional laboratories.

Hence, rapid point of care testing (on-farm, or in a local veterinary practice) have been shown to be able - with appropriate interpretation guidelines - to provide reliable first hand results for differentiation in growth from no growth, gram-positive from gram-negative growth (28) and gross identification of pathogen groups (genus level) (29, 30) even when performed by non-microbiologists.

Several studies evaluating on-farm rapid culture testing for clinical mastitis cases, show that the use of an on-farm culture system to guide the strategic treatment of clinical mastitis decreased intra-mammary antibiotic use by 25–50%, without significant negative impact on long term outcomes (e.g., bacteriological cure, subsequent retreatment risk, somatic cell count or culling risk) (31, 32). Also, on-farm culturing of milk at the end of lactation has been shown to have effect on reducing antibiotic treatment for drying off (33).

Several studies have been performed in the last two decades, evaluating different rapid culture systems worldwide, mostly focusing on clinical Mastitis (6, 28, 29, 32–34).

To our knowledge, no study has been performed analyzing subclinical and clinical cases of mastitis with the two rapid culture systems Selma Plus and Dipslide, available in Switzerland.

The aim of this study was to assess the agreement between test results from two accredited commercial laboratories and two point of care tests and to estimate the diagnostic test accuracies with a BLCM, using 509 single quarter milk samples from cows with clinical and subclinical mastitis. Additionally, we wanted to assess if the presence of a mixed infection is associated with changes in the prevalence or the diagnostic test sensitivities of the two point of care tests.

## Materials and methods

### Study population and samples

Analyzed milk samples (*n* = 509) originated from three different pools:

1. Pool 1: 45 dairy herds in different parts of Switzerland, enrolled in regular herd health visits from the herd health unit Vetsuisse Faculty, University of Bern.
2. Pool 2: Two private veterinary practices in the rural area of Switzerland.
3. Pool 3: Samples from a specific area of Switzerland (ReLait Project, Canton Fribourg) including 63 dairy farms.

Milk samples were collected aseptically either by the farm veterinarian (Pool 1,2,3) or by trained dairy producers (Pool 3) from cows with subclinical and clinical mastitis. The procedure of collecting the samples was performed according to the guidelines recommended by the National Mastitis Council (24) after aseptically preparing the teat.

Clinical mastitis was defined as milk that appeared abnormal with or without other local or systemic signs of inflammation. Subclinical mastitis was detected when the cow had a composite somatic cell count (SCC) higher than 150'000 cells/ml in the recent milk recording. In this case, California Mastitis Test was performed to identify the affected quarter.

Collected samples were refrigerated and sent directly (Pool 1) or *via* courier (Pool 2&3) to the laboratory within 12 h. Samples from pool 3 were analyzed in the external laboratory (standard culture, Labor Zentral) before being frozen and sent to the University Laboratory (ZOBA) for further analysis with the mastitis rapid point of care kits.

### Standard laboratory culture

Standard laboratory culture was performed by two accredited laboratories in Switzerland (ZOBA, Vetsuisse Faculty University of Bern, Switzerland and Labor Zentral, Geuensee, Switzerland) according to the following procedure:

#### Labor zentral

One loop of whole of milk (about 10μL) was streaked on a blood agar plate (blood agar with aesculin, Oxoid AG, Pratteln Switzerland), a chromID CPS Agar (Bio Mérieux SA, Petit Lancy, Switzerland) and on a Briliance Staph. agar (Oxoid AG, Pratteln, Switzerland) each.

##### ZOBA

To allow for better comparability in the framework of the present study, both the culture using whole milk as well as cultures of milk sediment, which represents the routine diagnostic procedure for the ZOBA laboratory, were performed. For sedimentation, milk tubes were centrifuged 10 min at 3000 rpm and the supernatant discarded. One loop full of whole milk and one loop full of milk sediment (each about 6μL) were streaked separately on a blood agar plate (Trypticase Soy Agar II with 5% Sheep Blood, BD) and on a Brolac agar plate (ThermoFisher, Oxoid AG, Pratteln, Switzerland) each. The results of the whole milk culture were taken as the standard culture to allow better comparability. The results of the milk sediment of the laboratory ZOBA were used only for one part of the BLCA of *Strept. uberis* in this study (see **Table 3**).

In both laboratories, blood agar plates were incubated at 37°C in a 5% CO_2_ atmosphere and Brolac plates were incubated aerobically. After 16 to 24 h colonies were identified with Maldi-Tof MS (MALDI Biotyper, Bruker, Fällanden, Switzerland).

### Point of care tests

#### Selma plus (SVA, National Veterinary Institute, Sweden)

The Selma plus plate is an agar plate divided in four sectors with different selective media: Bovine blood agar with esculin where gram-positive bacteria, gram-negative bacteria, yeast and algaewill grow; MacConkey agar, where only gram-negative bacteria grow; Mannitol agar, where staphylococci and enterococci grow and beta-glucuronidase (PGUA) agar for identification of *Escherichia coli. One* loop of the milk sample (about 10 μL) was spread on each agar field according to the producer's instruction. Isolates were classified as belonging to one of the following 11 species or species groups*: non-aureus Staphylococci* (NAS), *Staphylococcus aureus (S. aureus), Streptococcus spp., Enterococcus spp., Trueperella (T.) pyogenes, Escherichia coli (E. coli), Klebsiella, Candida*, other gram-negative, no growth and contaminated (CON).

##### Dipslide (AxonLab AG, Baden-Dätwil, Switzerland)

The Dipslide test tube (Dipslide) contains three different media for culturing mastitis causing pathogens: Brain-heart infusion (Medium 1 = BHI-Agar, PVP, pH 7.2), where gram-positive and gram-negative bacteria and yeast grow; Chromogenic Media for detection of gram-positive bacteria (Medium 2 = Chromogenic Substrate + Supplement, PVP, pH 7); Chromogenic Media for detection of gram-negative bacteria (Medium 3 = Chromogenic Substrate + Supplement, PVP, pH 7). A non-specified amount of whole milk (Min. 2 ml, Max. 10 ml) of the sample was spilled over both sides of the test kit (side 1 for medium 1, side 2 for media 2 and 3), making sure all media were poured with milk. Isolates were classified into following 10 categories: *Staphylococcus aureus (S. aureus), Streptococcus uberis (S. uberis), Enterococcus, Escherichia coli (E. coli), Klebsiella, Candida* spp., other gram-negative, other gram-positive, no growth and contaminated.

Two farm animal clinic veterinarians (residents, working at the Herd Health Unit, Vetsuisse Faculty, University of Bern) with no specific experience in microbiological culturing methods performed all laboratory procedures of the rapid point of care detection kits. The initial 50 milk samples were analyzed as training to adopt the test kits and the interpretations, and have not been added to our data. After this initial training, all results were interpreted according to the accompanying interpretative guidelines from the test kits by either one of the veterinarians.

### Qualification and interpretation of identified pathogens

Both, the Selma plus plates and Dipslide tubes were incubated at 37°C for 16–24 h. The first reading was preformed after 16–24 h. If there was no growth, the test was incubated for another period of 24 h.

The threshold for growth - and therefore considering a quarter infected - was set according to the definition of Dohoo et al. (25) at 100 CFU/ml equal one single colony from a 0.01 ml milk sample. Exception from this definition was made in the group of NAS, where also a borderline threshold of 100 CFU/ml was chosen, instead of the proposed 200 CFU/ml by the authors.

This definition was coherent between the on-farm culture and one laboratory-based system (Labor ZOBA, University of Bern, Switzerland), but slightly differed in the standard culture performed by the commercial laboratory (Labor Zentral, Geuensee, Switzerland). I.e., a single colony of a major pathogen was defined as intramammary infection for all culture systems, however a single colony of other pathogens (such as NAS) was defined as an infection for the on-farm culture and ZOBA, whereas three or more colonies were required for classifying as infection in the laboratory protocol of Labor Zentral.

Single quarter samples were categorized as contaminated (CON), if three or more morphologically different colonies were detected [according to NMC Handbook (35)] or no pathogenic bacteria could be isolated due to strong growth of several different bacterial species.

In the case of growth of two morphologically different bacteria, these were each classified according to the guidelines of the test kits and included in the results as mixed infection.

### Data analysis

The processing of milk samples and collection of data was carried out in the period from February to November 2019. The data cleaning, processing and categorization was done with Excel 2016 (36) and statistical analysis was performed with R (37). The data is available in the Supplementary material S1, S2. To describe the proportion of positive samples, 95 % binomial confidence intervals, following Jeffreys approach, were obtained with the command BinomCI() from the package DescTools (38). To assess if the proportions of positive test results differed between the three tests (pairwise) Mc Nemar's chisquare test was used. The agreement between test results of all three diagnostic approaches was obtained with Cohen's kappa available in the psych package (39). A Cohen's Kappa coefficient (κ) close to 1 indicates a good agreement, κ close to 0 indicates a poor agreement.

For bacteria with an apparent prevalence of at least 10%, Bayesian latent class models (BLCM), encompassing three tests (Dipslide, Selma plus and culture) in one population, were used to estimate diagnostic test accuracies of all three diagnostic tests (40). Solely, for *S. uberis*, the apparent prevalence was higher than 10% for samples tested by Dipslide and culture. All samples identified aesculin-positive *Streptococci* by Selma plus were assumed to be *S. uberis*.

The meaning of the latent class is presence or absence of specific bacteria.

Since the specificity of culture is assumed to be very high, the specificity of culture was fixed to 1 in the model (deterministic constraint). To assess the influence of conditional dependencies between the sensitivities, all two-way conditional dependencies or covariances were first included separately and then in all possible combinations. A model with a potential conditional dependency between the specificity of both rapid point tests was also included. In the absence of evident covariances [i.e., 95% credibility intervals including 0 and no substantial decrease in DIC (41)], the covariances were set to 0. Non-informative beta priors (1,1) were chosen for all sensitivities and the specificities of Selma plus and Dipslide. The model code is available in the Supplementary material S3. For the prevalence, using beta buster, an informative beta prior was used assuming (95% sure that the prevalence was smaller than 50% with a mode at 30%). This prior was based on the expert opinion of one of the Co-authors based on descriptive data of a study in progress (Sommer et al. in preparation). A sensitivity analysis was performed by varying the informative prior for the prevalence and using weakly informative priors beta (2,1) for the diagnostic test accuracies. For a subset of the data, where test results from samples analyzed from the sedimented milk, an additional BLCM analysis with tests results from Dipslide, culture and culture based on the sediment was performed.

Additionally, models allowing for a mixed infection to be included as a covariate for prevalence or sensitivity of Dipslide and Selma plus were also tested. The latent class models were fitted using Markov Chain Monte Carlo (MCMC) simulation by using the free statistical software JAGS (42) and the R packages runjags (43) and coda (44). For each model, three chains of the Gibbs sampler were run independently from different starting points of 100 000 iterations after a burn-in of 5,000 and a thinning of 10. The behavior of the MCMC chains was monitored through the plotting of the posterior trace plots to identify potential converging problems. The output files from the Gibbs sampler were analyzed through the package coda (44) calculating the potential scale reduction factor. We followed the STARD-BLCM guidelines (45), (Supplementary material S4).

## Results

### Descriptive results of microbiological analysis and test agreement beyond chance

A total of 509 single quarter milk samples were enrolled in the study. From those 509 samples, information about clinical presentation was available for 287 samples, with 108 cases (38%) classified as clinical mastitis and 179 (62%) cases as subclinical mastitis. In 222 individual quarter samples, no information on the clinical appearance could be determined. This high proportion is due to the fact, that the clinical appearance of mastitis was not consistently noted on the laboratory anamnesis sheet.

Pool 1 and 2 contributed 237 samples, while 73 samples came from Pool 3.

Microbial growth was isolated from 78, 89, and 88% of single quarters with clinical and subclinical mastitis for whole milk culture, Dipslide and Selma plus, respectively (Table 1).

**Table 1:** Descriptive statistics and Cohen's kappa agreement for 509 single quarter milk samples collected from 509 Swiss dairy cows suffering from clinical and subclinical mastitis.

**Pathogen**	**Test**	**Frequencies [*n =* 509]**		**Agreement (Cohen's Kappa;** κ**)**		
		**Positive *n***	**% [95% CI]**	**Dipslide - culture**	**Selma plus- culture**	**Dipslide – Selma plus**
Negative/No Growth	Culture	113	22.2 [18.8;26.0]	0.49 [0.4;0.59] s^6^	0.54 [0.45;0.63] s	0.82 [0.74;0.9] ns^6^
	Dipslide	56	11.0 [8.5;13.9]			
	Selma plus	63	12.3 [9.7;15.4]			
Gram positive ^1^	Culture	329	64.6 [60.4;68.7]	0.57 [0.49;0.64] s	0.59 [0.52;0.67] ns	0.79 [0.72;0.85] s
	Dipslide	376	73.9 [69.9;77.5]			
	Selma plus	378	74.3 [70.3;77.9]			
*Staphylococcus aureus*	Culture	27	5.3 [3.6;7.5]	0.48 [0.3;0.65] ns	0.72 [0.56;0.87] s	0.45 [0.25;0.65] ns
	Dipslide	21	4.1 [2–6;6.1]			
	Selma plus	17	3.3 [2.0–5.1]			
*Streptococcus spp*.	Culture	177	34.2 [30.2;38.4]	NA	0.58 [0.51;0.66] s	NA
	Dipslide	NA^5^	NA			
	Selma plus	185	36.3 [32.3;40.6]			
*Streptococcus uberis*	Culture	139	27.3 [23.6–31.2]	0.53 0.45;0.61 ns	NA	NA
	Dipslide	158	31.0 [27.1;35.1]			
	Selma plus	NA^5^	NA			
*Enterococcus*	Culture	22	4.3 [2.81;6.35]	NA^4^	0.21 [0.003^4^;0.42] s	0.36 [−0.009^4^;0.72] ns
	Dipslide	6	1.2 [0.49;2.41]			
	Selma plus	5	1.0 [0.36;2.14]			
Minor pathogens (NAS + *Corynebacterium spp*.)^3^	Culture	120	23.6 [20.0;27.4]	NA	0.48 [0.41;0.55] s	NA
	Dipslide	NA^5^	NA			
	Selma plus	216	42.4 [38.2;46.8]			
*Trueperella pyogenes*	Culture	10	1.9 [1.01;3.45]	NA	0.46 [0.12;0.79] s	NA
	Dipslide	NA^5^	NA			
	Selma plus	3	0.5 [0.16;1.56]			
Gram negative ^2^	Culture	40	8.6 [6.4;11.3]	0.49 [0.35;0.63] ns	0.66 [0.54;0.78] ns	0.7 [0.58;0.81] ns
	Dipslide	39	7.7 [5.6;10.2]			
	Selma plus	44	8.6 [6.4;11.3]			
*Escherichia coli*	Culture	28	5.5 [3.77;7.74]	0.66 [0.51;0.82] ns	0.81 [0.7;0.93] ns	0.75 [0.61;0.88] ns
	Dipslide	22	4.3 [2.81;6.35]			
	Selma plus	28	5.5 [3.77;7.74]			
*Klebsiella*	Culture	4	0.7 [0.26;1.86]	NA	0.17 [−0.14^4^;0.48] ns	NA
	Dipslide	NA^5^	NA			
	Selma plus	7	1.8 [0.62;2.68]			
*Candida*	Culture	4	0.8 [0.27;1.86]	NA	0.86 [0.58;1.0] ns	NA
	Dipslide	0	0 [0;0.49]			
	Selma plus	3	0.6 [0.17;1.56]			
Contaminated	Culture	34	6.7 [4.75;9.11]	0.44 [0.28;0.61] ns	0.43 [0.28;0.59] ns	0.79 [0.67;0.91] ns
	Dipslide	25	4.9 [3.28;7.05]			
	Selma plus	30	5.9 [4.09;8.19]			
Not identifiable	Culture	0	0.0	NA	NA	NA
	Dipslide	26	5.1 [3.44;7.28]			
	Selma plus	11	2.2 [1.15;3.71]			

^1^Gram positive: **Dipslide**: Gram positive (all unspecific growth on Medium 2), *Staphylococcus aureus, Streptococcus uberis, Enterococcus*; **Selma plus:** NAS, *Staphylococcus aureus, Streptococcus spp., Enterococcus, T. pyogenes*; **Culture:** NAS, *Staphylococcus aureus, Streptococcus spp., Enterococcus, T. pyogenes*, other Gram positive. ^2^Gram negative**: Dipslide**: *E. coli, Klebsiella*, other Gram negative (growth on Medium 3); **Selma plus:** *E. coli, Klebsiella*, other Gram negative (general growth on MacConkey agar); **Culture:** *E. coli, Klebsiella*. ^3^Minor pathogens: **Selma plus**: NAS; **Culture**: NAS + *Corynebacterium spp*. ^4^Negative Kappa or values close to 0 indicate that the number of agreement is lower than the expected number by chance. ^5^Unable to categorize the growth into one of the groups according to the manufacturer's guidelines. ^6^“s” and “ns” indicate if Mc Nemar's Chisquare test gave a significance or a non-significant test result (*p* < 0.05), respectively.

Overall, gram-positive bacteria were detected in 65–75% of all samples, with *Streptococcus* spp. as the main isolated bacteria. Gram-negative growth appeared in 8–9%, with *Escherichia coli* being responsible for more than half of the gram-negative cases.

No bacterial growth was recovered in 113, 56, and 63 samples, referring to 22, 11, and 12% for culture of whole milk, Dipslide and Selma plus, respectively.

The two rapid culture test kits yielded 26 and 11 non-identifiable results for Dipslide and Selma plus, respectively. The higher number in Dipslide is due to the fact, that all colonies, growing only on Medium 1 (BHI Medium), were not identifiable according to the interpretation guidelines of the test. The category “not identifiable” includes mainly colonies, that should have been recognized according to the test instructions, but could not be assigned on the basis of the morphology/appearance of the colony (e.g., growth on atypical media, unusual discoloration). Furthermore, bacteria such as *Serratia spp*. (*n* = 2), *Pasteurella multocida* (*n* = 1) or *Citrobacter krusei* (*n* = 1) which could not be identified according to the guidelines, but also *Proteus* spp. (*n* = 1), which is listed in the guidelines, but could not be identified, are included in this category.

Cohen's Kappa coefficient (κ) showed a wide range of agreement between negative **κ** values (*Enterococcus* Dipslide vs. culture) or low **κ** values (*Klebsiella* spp.; Selma plus vs. culture), indicating poor agreement between the test results (Table 1). Examples for a favorable agreement between all tests were detected for *Escherichia coli*, i.e., *(***κ** 0.66–0.81) for Selma plus plate compared to culture.

### Bayesian latent class models

BLCM were performed solely for *Streptococcus uberis*, because this was the only pathogen with proportion of at least 10% of the samples being positive. For Selma plus all S*treptococcus* samples were considered as *Streptococcus uberis*. Based on posterior trace plots and all potential scale reduction factors being below 1.1, all models converged. Regarding the 95% credibility intervals (CrI) and the deviance information criterion (DIC), the best model was a model including a covariance between the sensitivities of Dipslide and Selma plus. The results, including the posterior means and 95% credibility intervals, of both the independence model and the model with a covariance between the sensitivities of Dipslide and Selma plus, are displayed in Table 2. Including a covariance led to lower test accuracies. Including further covariances between the sensitivities did not alter the posterior estimates substantially. To assess if the prior of the prevalence (“being 95% sure, that the true value was below 50% with a mode at 30%”) affected the obtained posterior values, a sensitivity analysis was performed by varying the beta priors from assuming the true value to be below 30% with a mode of 10%, below 40% with a mode of 20% and so on, until below 90% with a mode of 70%. Additionally, a non-informative prior with a beta (1,1) for all parameters - and weakly informative priors with a beta (2,1) for the diagnostic test accuracies - were considered. None of the mean posterior values differed substantially from the initial model when varying the priors for prevalence or including weakly informative priors (Supplementary material S5). The largest difference found was a posterior prevalence mean of 42.2 compared to 41.0% indicating that the chosen informative prior had a negligible effect on the posterior distribution.

**Table 2:** Results of the final BLCMs for *Streptococcus uberis* encompassing three tests (Dipslide, Selma plus, and whole milk culture) in one population, to estimate diagnostic test accuracies of all three diagnostic tests using 509 milk samples collected from Swiss dairy cows suffering from clinical or subclinical mastitis.

	**Model**	**Model**	**Model**	**Model**	**Model**
**Estimates**	**independence**	**with covs**	**covariate on prevalence**	**covariate on Se Dipslide**	**covariate on Se Selma plus**
	**mean [95% CrI]**	**mean [95% CrI]**	**mean [95% CrI]**	**mean [95% CrI]**	**mean [95% CrI]**
Prevalence	42.2 [37.0;47.2]	41.1 [36.0;46.5]	-	41.5 [36.0;46.9]	41.8 [36.5;47.7]
			Prevalence	Sensitivity Dipslide	Sensitivity Selma
Posterior of interest Cov = 1	-	-	9.5 [1.7;22.8]	63.8 [44.8;77.4]	54.5 [35.4;73.4]
Posterior of interest Cov = 0	-	-	43.9 [38.3;49.9]	70.6 [62.0;78.5]	75.0 [66.7;82.2]
Se _Dipslide_	71.1 [63.9;78.4]	69.5 [61.7;77.2]	68.9 [60.8;77.0]	-	68.3 [60.1;76.4]
Se _Selma plus_	76.2 [69.9;82.4]	71.8 [64.6;79.2]	71.7 [64.4;79.1]	71.7 [64.2;79.3]	-
Se _Culture_	64.0 [56.1;71.5]	59.3 [52.9;66.0]	59.0 [52.2;65.4]	58.5 [51.2;65.6]	57.9 [51.2;65.3]
Sp _Dipslide_	98.4 [96.2;1]	95.1 [92.1;98.0]	95.0 [92.0;97.8]	95.8 [92.5;99.0]	95.5 [92.3;98.4]
Sp _Selmaplus_	93.2 [88.6;97.9]	88.5 [84.4;92.8]	88.6 [84.3;93.0]	88.8 [84.5;93.1]	90.0 [85.4;94.6]
Sp _Culture_	Fixed to 1	Fixed to 1	Fixed to 1	Fixed to 1	Fixed to 1
Covs	-	0.07 [0.05;0.09]	0.07 [0.05;0.09]	0.07 [0.05;0.08]	0.06 [0.04;0.08]
Intercept	-	-	−0.245 [−0.48; −0.001]	0.886 [0.48;1.29]	1.10 [0.69;1.53]
Slope	-	-	−2.19 [−3.65; −0.79]	−0.32 [−1.1;0.34]	−0.92 [−1.83;0.017]
DIC	1,539.9	1,477.5	1,464.7	1,478.6	1,475.3

All samples identified as *Streptococcus* by Selma were assumed to be *Streptococcus uberis*. The 95% credibility intervals of the independence model, the model with conditional dependency between sensitivities of Selma plus and Dipslide and models including as a covariate a mixed infection for prevalence and the sensitivities of Dipslide and Selma plus are shown.

**Figure 1:**
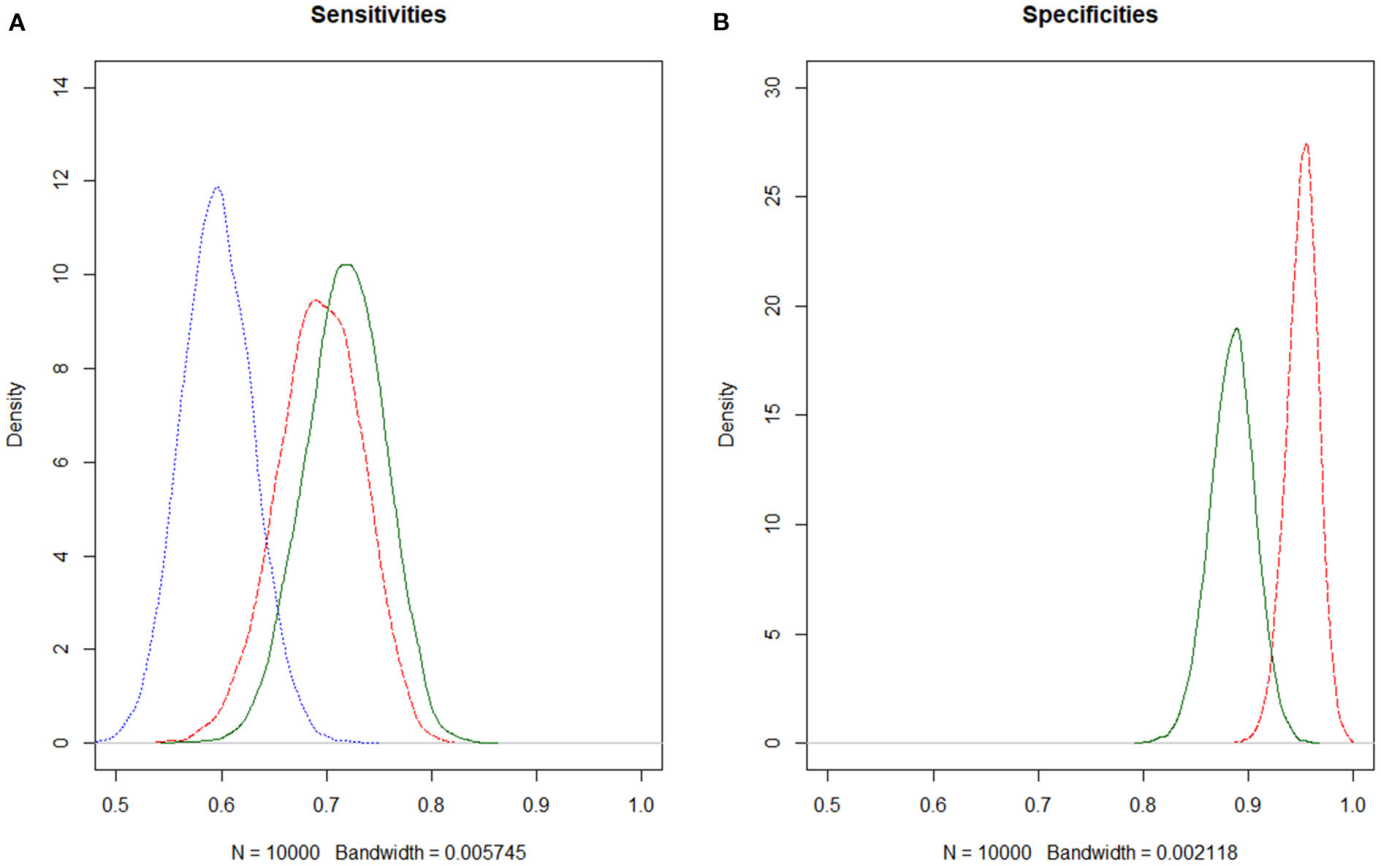
**(A,B)** Estimating diagnostic test accuracies for *S. uberis* of Selma plus, Dipslide and culture, using Bayesian latent class models. Dipslide, red dashed; Selma plus, green solid; Culture, blue dotted. Since the specificity of culture in general is assumed to be very high, the specificity of culture was fixed to 1 in the model.

When allowing for the covariate “mixed infection” in the model to have an impact on either the prevalence or the sensitivities of either the Dipslide or Selma plus, an effect was only seen for prevalence. Here a substantial decrease in DIC as well as a 95% credible interval of the regression coefficient not including 0 was found (on the log odds scale). The prevalence of *S. uberis* in the presence of a mixed infection was 9.5% with a 95% CrI [1.7;22.8] and 43.9% [38.3;49.9] in the absence of a mixed flora. Although the sensitivities of both Dipslide and Selma plus were reduced in the presence of a mixed infection, their corresponding credible intervals of the regression coefficient did contain 0 and the sensitivity intervals were overlapping (see Table 2 and Figures 1A,B).

When analyzing a subset of the data set for which culture was performed based on sedimentation (*n* = 434, comprising 118, 105, and 136 positive samples detected by culture with sedimentation, culture of whole milk and Dipslide), the sensitivity of culture of the milk sediment after centrifugation was considerably higher compared to culture of whole milk (Table 3). Compared to the analysis with the complete data set, all sensitivities are higher, whereas specificity of Dipslide and prevalence were lower.

**Table 3:** Outcomes of the final BLCM evaluating test accuracies for *Streptococcus uberis* of a subset of the data including Dipslide, culture of whole milk and culture of milk sediment.

	**Model**			
**Estimates**	**independence**		**With covs**	
	**posterior mean**	**95% CrI**	**posterior mean**	**95% CrI**
Prevalence	28.5	[24.2;32.6]	28.9	[24.6;33.1]
Se _Dipslide_	75	[68.4;83.3]	74.7	[66.9;82.2]
Se _Culturewholemilk_	84.9	[78.3;90.9]	83.6	[76.7;83.8]
Se _Culture.sediment_	95.3	[91.2;98.8]	93.9	[89.0;98.3]
Sp _Dipslide_	86.2	[82.2;89.9]	86.3	[82.4;90.1]
Sp _Culturewholemilk_	Fixed to 1	-	Fixed to 1	-
Sp _Culture.sediment_	Fixed to 1	-	Fixed to 1	-
Covs _Culture/Culture.sed_	-	-	0.004	[−0.003;0.014]
DIC	1,038.0		1,039.7	

## Discussion

This study was carried out to assess the agreement beyond chance between test results from reference laboratories and two point of care tests and to estimate the test accuracies with a BLCM, using 509 single quarter milk samples from cows with clinical and subclinical mastitis.

The agreement (κ) when comparing two tests varied greatly depending on the bacterial group or bacterial species.

High disagreement in pathogen identification was observed in the *Enterococcus* specie*s*. Differentiation of *Streptococcus, Enterococcus* and *Lactococcus* based on colony morphology only is not possible (46). That is why, both test kits included differentiation aids for correct identification of *Streptococcus spp*. and *Enterococcus spp*.. For *Escherichia coli (E. coli)*, κ is best for Selma plus compared to standard culture of whole milk in an accredited laboratory). Among gram-negative bacteria, *E. coli* is mainly responsible for clinical mastitis (47). With the ongoing efforts of evidence-based antibiotic use, mastitis caused by Gram negative bacteria does not benefit from antibiotic therapy (8, 48) and therefore accurate diagnosis is important for targeted use.

In bacteria and bacterial groups occurring in a higher prevalence, agreements are basically moderate to substantial. Negative or very poor κ occur in bacteria with a low prevalence in the study (e.g., *Enterococcus, Klebsiella*). In the case of *enterococci*, the reason may be that *enterococci* colonies on Dipslide were very similar in color to streptococci and therefore misclassified. The κ value is influenced by the prevalence and in this case basically not interpretable.

Since the definition of a contaminated sample was identical for all methods the κ for “contaminated sample” Selma plus-Dipslide showed a high agreement. It should be noted that the rapid culture tests only have small sectors where a relatively large amount of milk is spread. The exact morphological differentiation of growing colonies is therefore sometimes difficult. In standard culture, where one loop of milk is spread out per plate, the classification of individual colonies is easier and thus also more reliable. In addition, there is also an influence of the subjectivity of the evaluator, as the interpretation of what grows or does not grow on the culture plate depends much on the experience and training of the person evaluating the culture plate (49).

While κ only gives us information about the agreement between two test results, the BLCM was performed to determine the diagnostic test accuracies in the absence of a gold standard.

Based on BLCMs for *S. uberis*, the sensitivities of both Dipslide and Selma plus were higher than standard culture of whole milk in an accredited laboratory. Presumably different inoculums of milk in all three tests significantly influenced general growth rate, as a higher inoculum of milk is associated with a higher growth rate (50). Another study evaluating rapid culture tests also found similar results (31). Milk inoculum was highest and not standardized in Dipslide (2–10 ml per test). The BLCM analysis was carried out for *S. uberis*, because of the high prevalence and its current importance in practice. *S. uberis* is the most important *Streptococcus* species isolated in bacteriological culturing from mastitis milk samples (12). Furthermore, *S. uberis* mastitis cases profit from an extended antibiotic therapy (51). Therefore, out of the perspective of a clinician, it is crucial to know if a *S. uberis* is involved or not. Still, a major limitation of the study must be considered before interpreting the BLCM results, since Selma plus plate diagnoses Streptococcus at the genus level, only. All samples with a positive result of aesculin-positive *Streptococci* by Selma plus plate were considered in the BLCM as *S. uberis* based on the perspective of a veterinary practitioner for clinical decision-making considering *S. uberis* as a worst-case scenario.

The two point of care tests displayed a specificity above 90%, while the specificity of culture combined with MALDI-TOF species identification was fixed to 100%. The slight lower Sp for Selma plus compared to Dipslide is due to the limitation, as there were also other S*treptococci* species than *S. uberis* included in the analysis for Selma plus.

When including the covariate of mixed infection, the prevalence of *S. uberis* in the presence of a mixed infection was 9.5% and 43.9% in the absence of a mixed flora. Several studies imply a protective factor in quarters already infected with a minor pathogen (e.g., NAS or *Corynebacteria*) for infection with a major pathogen (e.g., *S. uberis*) (52–54) which could be a possible explanation for our findings. However, this hypothesis has been refused by other studies (55, 56) or could neither be determined as a risk factor nor as a protective factor for mastitis caused by major pathogens (57). Another factor may be inhibited growth of *S. uberis* in the presence of other bacteria in the milk sample due to inhibitory substances produced by minor pathogens or increased SCC in the milk (58, 59).

When using a subset of the data and including solely Dipslide and culture of whole milk as well as culture based on the sedimented samples, the sensitivity of the latter one was considerably higher compared to the samples analyzed without a sedimentation step. Also, Se of both culture methods are higher compared to Dipslide. Additional to the fact that the sediment might contained a higher density of bacteria, there are several potential reasons to explain this difference. The analyzed data is a subset of the whole data set, and sensitivities and specificities might change across populations (60). The data set just contained results from culture of milk sediment performed by one of the two laboratories (only samples out of pool 1&2). The omission of samples from pool 3 leads to a different sample composition and this might as well be responsible for the difference.

Using BLCMs to evaluate diagnostic test accuracies in mastitis diagnostics has been shown to be a reliable method (6, 27, 49). In our study, limitations are due to test specific identification of specific pathogen not being uniform for all tests *(Streptococcus uberis)* and slight differences in the detailed sample setting under filed conditions.

### Implementation in practice

When adopting point of care culture system in local veterinary businesses or on-farm, considerations about accuracy, cost, shelf life, necessary storage conditions and ease of use have to be evaluated (34). Additionally, as a veterinary practitioner you have to make sure to meet the regulations of your country concerning biosafety and disposal of potentially hazardous waste that accumulates when bacterial culture is performed in veterinary practice. However, at a time when antibiotic reduction is in the focus, more diagnostics not only serve this goal, but also provide a source of revenue for veterinary practices to meaningfully compensate for fewer antimicrobial sales and treatments.

The two evaluated rapid culture systems showing promising results for categories like general growth, and rough classification of the bacteria. Selma plus plate and Dipslide showing good test accuracies for the *Streptococcus* on the genus level. However, both rapid culture systems have their drawbacks: Selma plus is not able to specify Streptococci to the species level, so we only assumed that aesculin – positive *Streptococci* were equal to *S. uberis*, whereas Dipslide yielded a high proportion of *S. uberis* that were misclassified as *enterococci* and vice versa.

If treatment protocols require a more detailed information regarding the presence or absence of any particular bacterial species, the rapid culture systems evaluated in the present work may not be the test of choice. Even though they can reliably recognize some individual bacterial species (e.g., *E. coli*), they both are limited in recognition of certain other bacteria species (e.g., *Enterococcus, Klebsiella)*. In the case of *Corynebacterium*, which is one of the most prevalent minor pathogens isolated from milk samples in Switzerland (12), neither Dipslide nor Selma plus plate specifically detect *Corynebacterium*. None of the two test systems was able to detect *Trueperella pyogenes*, or algae. In addition, discrimination of NAS from *S. aureus* was not straight forward using the two tests. As a conclusion, the test may not be suitable for analysis of milk samples collected from cows with subclinical mastitis. Standard laboratory culture in combination with MALDI-TOF is considered as the method of choice in these cases, and therefore, we recommend sending the milk sample to a professional microbiological laboratory. However, it might be time saving to initially culture the milk on the rapid test media and if a pure culture of not identifiably bacteria is present, to send a swab of the culture to a professional lab for species identification and/or resistance testing.

In case of acute clinical mastitis however, when classification of pathogens in gram-negative, gram-positive or no growth influences treatment decision, the two evaluated rapid point of care detections kits can be recommended as first line test.

## Conclusion

Therefore, we conclude, that the two point of care rapid culture systems Dipslide and Selma plus plate can provide an important base for decision-making on targeted treatment of clinical mastitis, especially when information on growth/no growth and a rough classification of the bacteria into groups have an impact on the treatment strategy. Both rapid culture tests showing good test accuracies for *S. uberis* using BLCM, taking into account that Selma plus is not able to identify Streptococci to the species level and that the samples contained only a low number of *streptococci* other than *S. uberis* and very few *enterococci*, reflecting the field situation in our study. Further studies need to be performed using datasets and tests with uniform and more standardized pathogen identification and with a larger number of samples containing the most relevant mastitis pathogens. In addition to reliable test results, decision making for mastitis treatment should include factors at cow and herd level. Despite the limitation of our study due to the different sample pools and the difference in the level of pathogen detection between the rapid tests, we suggest that rapid tests at the disposal of the cattle practitioners are used more frequently. They can bring added value for targeted treatment decisions particularly in cases of acute clinical mastitis and support the reduction of antimicrobial resistance.

## Data availability statement

The original contributions presented in the study are included in the article/Supplementary material, further inquiries can be directed to the corresponding author/s.

## Ethics statement

Ethical review and approval was not required for the animal study because For our study there was no ethical review/approval necessary since the samples taken from the animals were milk samples taken during routine visits for diagnostic purposes to assess the quality of milk. Written informed consent for participation was not obtained from the owners because these animals were enrolled in regular health visits from either the Vetsuisse Faculty, two private practitioners or the farms took part in a project (ReLait).

## Author contributions

DR, MBo, and SH designed, conceptualized the study, and wrote the initial draft. DR, MBu, and SK conducted the study (sampling, analysing, and data entry). SK provided critical feedback. All authors read, amended, and approved the manuscript.

## References

[1] Heikkilä AM Nousiainen JI Pyörälä S. Costs of clinical mastitis with special reference to premature culling. J Dairy Sci. (2012) 95:139–50. 10.3168/jds.2011-4321 22192193

[2] Pol M Ruegg PL. Treatment practices and quantification of antimicrobial drug usage in conventional and organic dairy farms in Wisconsin. J Dairy Sci. (2007) 90:249–61. 10.3168/jds.S0022-0302(07)72626-7 17183093

[3] Menéndez González S Steiner A Gassner B Regula G. Antimicrobial use in Swiss dairy farms: Quantification and evaluation of data quality. Prev Vet Med. (2010) 95:50–63. 10.1016/j.prevetmed.2010.03.004 20381180

[4] Erskine RJ Walker RD Bolin CA Bartlett PC White DG. Trends in antibacterial susceptibility of mastitis pathogens during a seven-year period. J Dairy Sci. (2002) 85:1111–8. 10.3168/jds.S0022-0302(02)74172-6 12086045

[5] Stevens M Piepers S Supré K Dewulf J de Vliegher S. Quantification of antimicrobial consumption in adult cattle on dairy herds in Flanders, Belgium, and associations with udder health, milk quality, and production performance. J Dairy Sci. (2016) 99:2118–30. 10.3168/jds.2015-10199 26778315

[6] Jones G Bork O Ferguson SA Bates A. Comparison of an on-farm point-of-care diagnostic with conventional culture in analysing bovine mastitis samples. J Dairy Res. (2019) 86:222–5. 10.1017/S0022029919000177 31038086

[7] Owens WE Ray CH Watts JL Yancey RJ. Comparison of Success of Antibiotic Therapy during Lactation and Results of Antimicrobial Susceptibility Tests for Bovine Mastitis. J Dairy Sci. (1997) 80:313–7. 10.3168/jds.S0022-0302(97)75940-X 9058273

[8] Roberson JR. Establishing treatment protocols for clinical mastitis. Vetery Clin North Am Food Animal Pract. (2003) 19:223–34. 10.1016/S0749-0720(02)00071-3 12682944

[9] Ruegg PL. Treatment of mastitis in lactating cows: New bugs, old drugs and changing expectations. Cattle Practice. (2014) 22:111–6. Available online at: https://scholar.google.com/scholar_lookup?author=PL+Ruegg+&publication_year=2014&title=Treatment+of+mastitis+in+lactating+cows%3A+new+bugs,+old+drugs+and+changing+expectations&journal=Cattle+Pract.&volume=22&pages=111-6

[10] Tenhagen BA Köster G Wallmann J Heuwieser W. Prevalence of mastitis pathogens and their resistance against antimicrobial agents in dairy cows in Brandenburg, Germany. J Dairy Sci. (2006) 89:2542–51. 10.3168/jds.S0022-0302(06)72330-X 16772573

[11] Pitkälä A Haveri M Pyörälä S Myllys V Honkanen-Buzalski T. Bovine mastitis in Finland 2001 - Prevalence, distribution of bacteria, and antimicrobial resistance. J Dairy Sci. (2004) 87:2433–41. 10.3168/jds.S0022-0302(04)73366-4 15328265

[12] Kretzschmar L van den Borne BHP Kaufmann T Reist M Strabel D Harisberger M. Mastitis-Management in Schweizer Milchviehbetrieben Mastitis-Management in Schweizer Milchviehbetrieben mit Eutergesundheitsproblemen. Band. (2013) 155:453–62. 10.1024/0036-7281/a000491 23919972

[13] Overesch G Stephan R Perreten V. Antimicrobial susceptibility of gram-positive udder pathogens Antimicrobial susceptibility of gram-positive udder pathogens from bovine mastitis milk in Switzerland. Band. (2013) 155:339–50. 10.1024/0036-7281/a000469 23732380

[14] Halasa T Huijps K Østerås O Hogeveen H. Economic effects of bovine mastitis and mastitis management: A review. Veter Quart. (2007) 29:18–31. 10.1080/01652176.2007.9695224 17471788

[15] Huijps K Lam TJGM Hogeveen H. Costs of mastitis: Facts and perception. J Dairy Res. (2008) 75:113–20. 10.1017/S0022029907002932 18226298

[16] Seegers H Fourichon C Beaudeau F. Production effects related to mastitis and mastitis economics in dairy cattle herds. Vet Res. (2003) 34:475–91. 10.1051/vetres:2003027 14556691

[17] Nielen M Schukken YH Brand A Deluyker HA Maatje K. Detection of Subclinical Mastitis from On-Line Milking Parlor Data. J Dairy Sci. (1995) 78:1039–49. 10.3168/jds.S0022-0302(95)76720-0 7622715

[18] Dürr JW Cue RI Monardes HG Moro-Méndez J Wade KM. Milk losses associated with somatic cell counts per breed, parity and stage of lactation in Canadian dairy cattle. Livest Sci. (2008) 117:225–32. 10.1016/j.livsci.2007.12.004

[19] Agristat Bundesamt für Satistik S. Statistik Milchverwertung. (2020)

[20] Urech E Puhan Z Schällibaum M. Changes in milk protein fraction as affected by subclinical mastitis. J Dairy Sci. (1999) 82:2402–11. 10.3168/jds.S0022-0302(99)75491-3 10575607

[21] Federal Department of Home Affairs. Verordnung des EDI über die Hygiene bei der Milchproduktion. admin.ch. (2013) 2005:1–14. Available online at: http://www.admin.ch/opc/de/classified-compilation/20051436/index.html (accessed November 26, 2022).

[22] Office FFS and V. Available online at: https://www.blv.admin.ch/blv/en/home/das-blv/strategien/nationale-strategie-antibiotikaresistenzen.html (accessed November 26, 2022).

[23] Ruegg PL. Investigation of mastitis problems on farms. Veter Clin North Am Food Animal Pract. (2003) 19:47–73. 10.1016/S0749-0720(02)00078-6 12682935

[24] National Mastitis Council. Laboratory Handbook on Bovine Mastitis. (1999).

[25] Dohoo IR Smith J Andersen S Kelton DF Godden S. Diagnosing intramammary infections: Evaluation of definitions based on a single milk sample. J Dairy Sci. (2011) 94:250–61. 10.3168/jds.2010-3559 21183035

[26] Nonnemann B Lyhs U Svennesen L Ann Kristensen K Klaas IC Pedersen K. Bovine mastitis bacteria resolved by MALDI-TOF mass spectrometry. J Dairy Sci. (2019) 102:2515–24. 10.3168/jds.2018-15424 30639010

[27] Holmøy IH Toft N Jørgensen HJ Mørk T Sølverød L Nødtvedt A. Latent class analysis of real time qPCR and bacteriological culturing for the diagnosis of Streptococcus agalactiae in cow composite milk samples. Prev Vet Med. (2018) 154:119–23. 10.1016/j.prevetmed.2018.03.019 29685435

[28] Jones M Hochhalter J Lago A Bey R Godden S. Validation of the Minnesota easy culture system II : results from in-lab tri-plate culture versus standard laboratory culture, and tri-plate inter-reader agreement. In: American Association of Bovine Practitioners Proceedings of the Annual Conference. (2006) 299–300.

[29] Viora L Graham EM Mellor DJ Reynolds K Simoes PBA Geraghty TE. Evaluation of a culture-based pathogen identification kit for bacterial causes of bovine mastitis. Veter Record. (2014) 175:89. 10.1136/vr.102499 25013087

[30] Malcata FB Pepler PT Zadoks RN Viora L. Laboratory-based evaluation of a simplified point-of-care test intended to support treatment decisions in non-severe bovine clinical mastitis. J Dairy Res. (2021) 88:170–5. 10.1017/S0022029921000303 33958019

[31] McDougall S Niethammer J Graham EM. Antimicrobial usage and risk of retreatment for mild to moderate clinical mastitis cases on dairy farms following on-farm bacterial culture and selective therapy. N Z Vet J. (2018) 66:98–107. 10.1080/00480169.2017.1416692 29241025

[32] Lago A Godden SM Bey R Ruegg PL Leslie K. The selective treatment of clinical mastitis based on on-farm culture results: I Effects on antibiotic use, milk withholding time, and short-term clinical and bacteriological outcomes. J Dairy Sci. (2011) 94:4441–56. 10.3168/jds.2010-4046 21854917

[33] Cameron M Keefe GP Roy JP Dohoo IR MacDonald KA McKenna SL. Evaluation of a 3M Petrifilm on-farm culture system for the detection of intramammary infection at the end of lactation. Prev Vet Med. (2013) 111:1–9. 10.1016/j.prevetmed.2013.03.006 23597619

[34] Lago A Godden SM. Use of rapid culture systems to guide clinical mastitis treatment decisions. Veter Clin North Am Food Animal Pract. (2018) 34:389–412. 10.1016/j.cvfa.2018.06.001 30316499

[35] National Mastitis Council (NMC). Laboratory Handbook on Bovine Mastitis. 3rd Editio. New Prague, MN, USA: NMC. (2017).

[36] Excel (2016) Microsoft Corporation.

[37] Team R Development Core. A Language and Environment for Statistical Computing. R Foundation for Statistical Computing. (2018) 2. Available online at: http://www.r-project.org (accessed November 26, 2022).

[38] Signorell A Ken A Andreas A Nanina A Tomas A. DescTools: Tools for descriptive statistics. R package version 099 18 (2016)

[39] Revelle W. Psych: Procedures for Personality and Psychological Research. Evanston, IL, USA: Northwestern University. (2017)

[40] Lewis FI Torgerson PR A. tutorial in estimating the prevalence of disease in humans and animals in the absence of a gold standard diagnostic. Emerg Themes Epidemiol. (2012) 9:1–8. 10.1186/1742-7622-9-9 23270542 PMC3558341

[41] Spiegelhalter DJ Best NG Carlin BP van der Linde A. Bayesian measures of model complexity and fit. J R Stat Soc Series B Stat Methodol. (2002) 64:583–616. 10.1111/1467-9868.00353

[42] Plummer M Clayton D. Estimation of Population Exposure in Ecological Studies. J R Statist Soc. (1996) 58:113–26. 10.1111/j.2517-6161.1996.tb02070.x

[43] Denwood MJ. runjags: An R package providing interface utilities, model templates, parallel computing methods and additional distributions for MCMC models in JAGS. J Stat Softw. (2016) 71:1–25. 10.18637/jss.v071.i09

[44] Plummer M Best N Cowles K Vines K. CODA: convergence diagnosis and output analysis for MCMC. R news. (2006) 6:7–11. Available online at: https://scholar.google.co.uk/scholar?as_sauthors=M+Plummer&as_q=CODA%3A+convergence+diagnosis+and+output+analysis+for+MCMC&as_occt=title

[45] Kostoulas P Nielsen SS Branscum AJ Johnson WO Dendukuri N Dhand NK. Standards for the reporting of diagnostic accuracy studies that use Bayesian latent class models. Prev Vet Med. (2017) 138:37–47. 10.1016/j.prevetmed.2017.01.006 28237234

[46] CDC: Center vor Disease Control and Prevention. Identification of Other Streptococcus Species: Streptococcus General Methods; Section II. (2006)

[47] Thomas V de Jong A Moyaert H Simjee S el Garch F Morrissey I. Antimicrobial susceptibility monitoring of mastitis pathogens isolated from acute cases of clinical mastitis in dairy cows across Europe: VetPath results. Int J Antimicrob Agents. (2015) 46:13–20. 10.1016/j.ijantimicag.2015.03.013 26003836

[48] Pyörälä S Kaartinen L Käck H Rainio V. Efficacy of two therapy regimens for treatment of experimentally induced escherichia coli mastitis in cows. J Dairy Sci. (1994) 77:453–61. 10.3168/jds.S0022-0302(94)76973-3 8182170

[49] Nyman AK Persson Waller K Emanuelson U Frössling J. Sensitivity and specificity of PCR analysis and bacteriological culture of milk samples for identification of intramammary infections in dairy cows using latent class analysis. Prev Vet Med. (2016) 135:123–31. 10.1016/j.prevetmed.2016.11.009 27931924

[50] Lam TJ van Wuijckhuise LA FP. Use of composite milk samples for diagnosis of Staphylococcus aureus mastitis in dairy cattle. J Am Vet Med Assoc. (1996) 208:1705–8. 8641956

[51] Hillerton JE Kliem KE. Effective treatment of Streptococcus uberis clinical mastitis to minimize the use of antibiotics. J Dairy Sci. (2002) 85:1009–14. 10.3168/jds.S0022-0302(02)74161-1 12018412

[52] Rainard P Poutrel B. Effect of naturally occurring intramammary infections by minor pathogens on new infections by major pathogens in cattle. Am J Vet Res. (1988) 49:327–9. 3282458

[53] Piepers S Opsomer G Barkema HW de Kruif A de Vliegher S. Heifers infected with coagulase-negative staphylococci in early lactation have fewer cases of clinical mastitis and higher milk production in their first lactation than noninfected heifers. J Dairy Sci. (2010) 93:2014–24. 10.3168/jds.2009-2897 20412915

[54] Schukken YH. Intramammary infections and risk factors for clinical mastitis in herds with low somatic cell counts in bulk milk. Veter Rec J Br Veter Assoc. (1989) 125:393–396. 10.1136/vr.125.15.393 2683341

[55] Hogan JS Smith KL Todhunter DA Schoenberger PS. Rate of environmental mastitis in quarters infected with Corynebacterium bovis and Staphylococcus species. J Dairy Sci. (1988) 71:2520–5. 10.3168/jds.S0022-0302(88)79840-9 3183146

[56] Parker KI Compton C Anniss FM Weir A Heuer C McOougall S. Subclinical and clinical mastitis in heifers following the use of a teat sealant precalving. J Dairy Sci. (2007) 90:207–18. 10.3168/jds.S0022-0302(07)72622-X 17183089

[57] Zadoks RN Allore HG Barkema HW Sampimon OC Wellenberg GJ Gröhn YT. Cow- and quarter-level risk factors for Streptococcus uberis and Staphylococcus aureus mastitis. J Dairy Sci. (2001) 84:2649–63. 10.3168/jds.S0022-0302(01)74719-4 11814021

[58] de Vliegher S Opsomer G Vanrolleghem A Devriese LA Sampimon OC Sol J. In vitro growth inhibition of major mastitis pathogens by Staphylococcus chromogenes originating from teat apices of dairy heifers. Vet Microbiol. (2004) 101:215–21. 10.1016/j.vetmic.2004.03.020 15223126

[59] Nascimento JDS Fagundes PC Brito MAVDP Netto Dos Santos KR Bastos MDCDF. Production of bacteriocins by coagulase-negative staphylococci involved in bovine mastitis. Vet Microbiol. (2005) 106:61–71. 10.1016/j.vetmic.2004.10.014 15737474

[60] Berkvens D Speybroeck N Praet N Adel A Lesaffre E. Estimating disease prevalence in a Bayesian framework using probabilistic constraints. Epidemiology. (2006) 17:145–53. 10.1097/01.ede.0000198422.64801.8d 16477254

