# Supplementary material for: Bayesian latent class models to determine diagnostic sensitivities and specificities of two point of care rapid tests (Selma plus, Dipslide) for the detection of Streptococcus uberis associated with mastitis in dairy cows
Source: Front Vet Sci. 2022 Dec 13;9:1062056. doi: 10.3389/fvets.2022.1062056 (PMC9792763; doi:10.3389/fvets.2022.1062056)
Supplement: Supplementary file 1 [file Data_Sheet_1.zip › S2.data.docx]

N <- 509

m.uberis <-

structure(c(0, 0, 0, 0, 0, 0, 0, 0, 1, 1,

0, 1, 1, 1, 0, 0, 0, 1, 0, 0, 0, 0, 0,

0, 0, 0, 0, 0, 0, 0, 0, 1, 1, 0, 0, 0,

0, 1, 0, 1, 0, 0, 1, 1, 0, 0, 0, 0, 1,

1, 1, 1, 0, 0, 0, 0, 0, 0, 1, 0, 1, 0,

0, 0, 1, 1, 0, 0, 0, 1, 1, 0, 0, 1, 0,

1, 0, 1, 0, 1, 0, 0, 0, 1, 1, 1, 0, 0,

0, 1, 1, 0, 0, 0, 0, 0, 0, 1, 0, 0, 0,

0, 0, 1, 0, 1, 0, 0, 0, 0, 1, 1, 1, 0,

0, 0, 0, 0, 1, 0, 0, 1, 0, 0, 0, 0, 0,

1, 0, 0, 0, 0, 1, 1, 0, 0, 0, 1, 1, 0,

0, 0, 0, 0, 1, 1, 1, 1, 0, 0, 1, 0, 1,

0, 0, 0, 0, 0, 1, 1, 0, 1, 0, 0, 0, 1,

0, 1, 1, 1, 1, 0, 1, 0, 0, 0, 0, 0, 1,

0, 0, 0, 0, 0, 0, 0, 0, 0, 0, 0, 1, 0,

0, 0, 0, 0, 0, 0, 0, 1, 1, 0, 1, 0, 0,

0, 0, 1, 1, 0, 1, 0, 0, 0, 0, 0, 0, 0,

1, 0, 1, 0, 0, 0, 0, 0, 0, 0, 0, 0, 0,

0, 1, 1, 1, 0, 0, 0, 0, 1, 0, 0, 1, 0,

0, 0, 1, 0, 1, 0, 0, 1, 0, 0, 0, 0, 1,

1, 0, 1, 1, 0, 0, 0, 0, 0, 0, 0, 1, 0,

0, 0, 0, 0, 0, 1, 0, 0, 0, 1, 1, 0, 1,

1, 0, 0, 0, 0, 0, 0, 1, 0, 0, 1, 1, 0,

1, 0, 0, 0, 0, 0, 0, 0, 0, 0, 1, 0, 1,

0, 1, 0, 0, 1, 0, 0, 1, 1, 1, 0, 0, 0,

0, 1, 0, 1, 0, 0, 0, 0, 0, 0, 0, 1, 1,

0, 0, 0, 1, 0, 0, 1, 1, 1, 0, 0, 0, 0,

0, 0, 0, 0, 0, 0, 1, 0, 0, 0, 0, 0, 0,

1, 0, 0, 1, 0, 0, 1, 0, 0, 1, 1, 1, 0,

0, 1, 0, 0, 0, 0, 0, 0, 0, 0, 1, 0, 1,

1, 1, 0, 0, 0, 0, 0, 1, 0, 0, 1, 0, 0,

1, 1, 0, 0, 0, 0, 0, 0, 0, 1, 1, 0, 0,

0, 0, 0, 0, 0, 1, 0, 0, 1, 0, 0, 0, 1,

0, 0, 1, 1, 0, 0, 1, 0, 0, 0, 1, 1, 0,

1, 1, 0, 1, 1, 0, 1, 0, 1, 0, 0, 0, 0,

0, 0, 0, 0, 0, 0, 0, 0, 1, 1, 1, 1, 0,

0, 0, 0, 1, 1, 1, 0, 1, 0, 0, 1, 0, 0,

0, 0, 0, 0, 0, 1, 0, 0, 1, 0, 1, 1, 0,

0, 0, 0, 0, 1, 0, 1, 1, 0, 0, 1, 0, 0,

1, 1, 0, 1, 0, 0, 1, 0, 0, 0, 0, 0, 0,

0, 1, 0, 1, 1, 1, 0, 0, 0, 1, 0, 0, 1,

1, 0, 0, 0, 0, 0, 0, 0, 0, 0, 1, 1, 0,

0, 0, 0, 1, 0, 1, 1, 0, 1, 1, 0, 0, 0,

0, 1, 1, 1, 1, 0, 0, 0, 0, 0, 0, 0, 1,

1, 0, 0, 0, 1, 0, 0, 0, 0, 1, 1, 0, 0,

1, 0, 1, 0, 0, 0, 1, 0, 0, 0, 1, 1, 1,

0, 0, 0, 1, 1, 0, 0, 0, 0, 0, 0, 1, 0,

0, 0, 0, 1, 1, 0, 1, 0, 0, 0, 0, 1, 0,

0, 0, 0, 1, 0, 0, 1, 0, 0, 1, 0, 0, 0,

0, 0, 1, 0, 0, 0, 0, 1, 0, 0, 0, 0, 0,

1, 0, 0, 0, 0, 0, 1, 1, 1, 1, 0, 0, 1,

0, 1, 0, 0, 0, 0, 0, 1, 1, 1, 1, 1, 0,

1, 0, 0, 1, 1, 1, 1, 0, 1, 0, 0, 0, 1,

0, 1, 0, 0, 0, 0, 0, 0, 0, 0, 0, 0, 0,

1, 0, 0, 1, 0, 0, 0, 0, 0, 1, 1, 0, 1,

1, 0, 0, 0, 1, 1, 1, 1, 0, 1, 1, 0, 0,

0, 0, 1, 0, 1, 1, 0, 0, 0, 0, 0, 0, 0,

0, 0, 0, 1, 1, 1, 0, 1, 0, 0, 0, 0, 0,

1, 0, 0, 0, 1, 0, 1, 0, 0, 1, 0, 0, 0,

0, 1, 1, 1, 1, 1, 1, 0, 0, 0, 0, 0, 0,

0, 0, 0, 1, 0, 0, 0, 0, 1, 0, 0, 1, 1,

0, 1, 1, 0, 0, 0, 0, 0, 0, 1, 0, 1, 1,

1, 0, 1, 0, 0, 0, 0, 0, 0, 0, 0, 0, 1,

0, 1, 1, 1, 0, 0, 1, 0, 0, 1, 1, 1, 1,

0, 0, 0, 1, 0, 0, 0, 0, 0, 0, 0, 0, 0,

0, 1, 0, 1, 1, 1, 0, 0, 1, 0, 1, 0, 1,

0, 0, 0, 0, 0, 0, 0, 0, 1, 0, 0, 0, 0,

1, 0, 1, 0, 0, 1, 0, 1, 1, 0, 0, 1, 1,

1, 0, 1, 1, 0, 0, 0, 0, 0, 0, 0, 0, 0,

0, 1, 1, 1, 0, 0, 1, 0, 0, 1, 0, 1, 1,

0, 0, 1, 1, 1, 1, 0, 0, 0, 0, 0, 1, 1,

1, 1, 1, 0, 1, 0, 0, 1, 0, 0, 1, 0, 0,

0, 0, 0, 0, 1, 1, 0, 0, 1, 0, 0, 0, 1,

1, 0, 1, 1, 0, 1, 1, 0, 1, 0, 1, 0, 0,

1, 0, 0, 0, 0, 0, 0, 0, 1, 0, 1, 1, 1,

1, 1, 0, 0, 0, 1, 1, 1, 0, 1, 1, 0, 1,

0, 0, 0, 0, 0, 0, 0, 1, 0, 0, 0, 1, 1,

1, 0, 0, 0, 0, 0, 1, 0, 1, 1, 1, 0, 1,

1, 0, 1, 0, 0, 1, 0, 0, 0, 0, 0, 0, 0,

0, 0, 0, 1, 0, 0, 0, 1, 0, 0, 0, 0, 0,

0, 1, 0, 0, 0, 0, 0, 0, 0, 0, 0, 0, 1,

1, 0, 0, 0, 0, 1, 0, 1, 0, 0, 1, 1, 0,

0, 0, 0, 1, 1, 0, 1, 0, 0, 0, 0, 0, 0,

0, 0, 0, 1, 0, 0, 1, 1, 0, 0, 0, 1, 0,

0, 0, 0, 0, 1, 0, 0, 0, 1, 0, 0, 0, 1,

1, 0, 0, 0, 0, 0, 1, 0, 0, 0, 0, 0, 0,

1, 0, 0, 0, 0, 0, 1, 0, 1, 0, 0, 0, 0,

0, 0, 0, 0, 0, 0, 0, 0, 1, 0, 0, 1, 0,

0, 0, 0, 0, 0, 0, 0, 0, 0, 1, 1, 0, 0,

0, 0, 0, 0, 0, 0, 0, 0, 0, 0, 1, 1, 0,

0, 0, 0, 1, 0, 0, 0, 0, 0, 1, 1, 1, 1,

1, 0, 1, 0, 0, 0, 0, 1, 1, 0, 0, 0, 0,

0, 0, 0, 1, 0, 0, 0, 0, 0, 1, 0, 1, 1,

0, 0, 1, 0, 0, 0, 0, 0, 0, 0, 0, 1, 1,

0, 1, 0, 0, 0, 0, 1, 0, 1, 1, 0, 1, 0,

0, 0, 0, 0, 1, 0, 1, 0, 0, 0, 0, 0, 0,

0, 1, 0, 1, 0, 0, 0, 0, 0, 0, 0, 0, 1,

1, 0, 1, 1, 0, 0, 1, 0, 0, 1, 0, 1, 0,

0, 0, 0, 1, 1, 0, 0, 1, 0, 0, 0, 0, 1,

1, 0, 0, 1, 0, 0, 0, 0, 0, 0, 0, 0, 0,

0, 1, 0, 1, 1, 0, 0, 0, 0, 1, 0, 1, 0,

0, 0, 1, 0, 1, 0, 0, 0, 0, 1, 0, 0, 0,

0, 1, 0, 1, 0, 1, 0, 0, 1, 0, 0, 1, 0,

1, 0, 0, 0, 0, 1, 0, 0, 0, 0, 0, 1, 0,

0, 0, 1, 1, 0, 1, 0, 0, 0, 0, 0, 0, 1,

0, 0, 0, 0, 0, 0, 0, 0, 0, 0, 1, 0, 0,

0, 0, 0, 0, 0, 0, 0, 1, 0, 0, 1, 0, 0,

0, 0, 0, 0, 0, 1, 1, 1, 1, 1, 1, 1, 1,

1, 1, 0, 0, 0, 0, 0, 0, 1, 1, 1, 0, 0,

0, 1, 0, 0, 1, 1, 1, 0, 0, 0, 0, 0, 0,

0, 1, 0, 0, 0, 0, 0, 0, 0, 1, 0, 0, 1,

0, 0, 0, 0, 0, 0, 0, 0, 0, 0, 1, 0, 0,

0, 1, 1, 0, 0, 1, 0, 1, 1, 0, 1, 0, 1,

0, 0, 0, 0, 0, 0, 0, 0, 0, 0, 0, 0, 1,

1, 1, 1, 1, 1, 0, 0, 1, 1, 1, 0, 1, 0,

0, 1, 0, 0, 0, 0, 1, 1, 0, 1, 0, 0, 0,

0, 0, 1, 0, 0, 1, 0, 1, 0, 0, 1, 1, 0,

0, 0, 0, 0, 0, 0, 0, 1, 0, 0, 0, 0, 0,

0, 0, 0, 0, 0, 0, 0, 0, 0, 0, 0, 0, 0,

0, 0, 0, 0, 0, 0, 0, 0, 0, 0, 0, 0, 0,

0, 0, 0, 0, 0, 0, 0, 0, 0, 0, 0, 0, 0,

0, 0, 0, 0, 0, 0, 0, 0, 0, 0, 0, 1, 1,

0, 0, 0, 0, 0, 1, 1, 1, 0, 0, 0, 0, 0,

0, 0, 0, 0, 0, 0, 0, 0, 0, 0, 0, 0, 0,

0, 0, 0, 0, 1, 0, 0, 0, 0, 0, 0, 0, 0,

1, 1, 0, 0, 0, 0, 0, 0, 0, 0, 0, 0, 1,

0, 0, 0, 0, 0, 0, 0, 0, 1, 0, 0, 0, 0,

0, 0, 0, 0, 0, 0, 0, 0, 1, 0, 0, 0, 0,

0, 0, 0, 0, 0, 1, 0, 0, 0, 0, 0, 0, 0,

0, 0, 0, 0, 0, 0, 0, 0, 0, 0, 0, 0, 0,

0, 0, 0, 0, 0, 0, 0, 0, 0, 0, 0, 0, 0,

0, 0, 0, 0, 0, 0, 0, 0, 0, 0, 0, 0, 0,

0, 0, 0, 0, 0, 0, 0, 0, 0, 0, 0, 0, 0,

0, 0, 0, 0, 0, 0, 0, 0, 0, 0, 0, 0, 0,

0, 0, 0, 0, 0, 0, 0, 0, 0, 0, 0, 0, 0,

0, 0, 0, 0, 1, 0, 0, 0, 0, 0, 0, 0, 0,

0, 0, 0, 0, 0, 0, 1, 1, 0, 0, 0, 0, 0,

0, 0, 0, 0, 0, 0, 0, 0, 0, 0, 0, 0, 0,

0, 0, 1, 1, 0, 1, 1, 0, 0, 0, 0, 0, 0,

0, 0, 0, 0, 0, 0, 0, 0, 0, 0, 0, 0, 0,

0, 0, 0, 0, 0, 0, 0, 0, 0, 0, 0, 0, 0,

0, 0, 0, 0, 0, 0, 0, 0, 0, 0, 0, 0, 0,

0, 0, 0, 0, 0, 0, 0, 0, 0, 0, 0, 0, 0,

0, 0, 0, 0, 0, 0, 0, 0, 0, 0, 0, 0, 0,

0, 0, 0, 0, 0, 0, 0, 0, 0, 0, 0, 0, 0,

0, 0, 0, 0, 0, 0, 0, 0, 0, 0, 0, 0, 0,

0, 0, 0, 0, 0, 0, 0, 0, 0, 0, 0, 0, 0,

1, 1, 0, 0, 0, 0, 0, 0, 0, 0, 0, 0, 0,

0, 0, 0, 0, 0, 0, 0, 0, 0, 0, 0, 0, 0,

1, 0, 0, 0, 0, 0, 0, 0, 0, 0, 0, 0, 0,

0, 0, 0, 0, 0, 0, 0, 0, 0, 0, 0, 0, 0,

1, 0, 0, 0, 0, 0, 0, 0, 0, 0, 0, 0, 0,

0, 0, 0, 0, 0, 0, 0, 0, 0, 0, 0, 0, 0,

0, 0, 0, 0, 0, 0, 1, 0, 0, 0, 0, 0, 0,

0, 1, 0, 0, 0, 0, 0, 0, 0, 0, 0, 0, 0,

0, 0, 0, 0, 0, 0, 0, 0, 0, 0, 0, 0, 0,

0, 0, 0, 0, 0, 0, 0, 0, 0, 0, 0, 0, 0,

0, 0, 0, 0, 0, 0, 0, 0, 0, 0, 0, 0, 0,

0, 0, 0, 0, 0, 0, 0, 0, 0, 0, 0, 0, 0,

0, 0, 0, 0, 0, 0, 0, 0, 0, 0, 0, 0, 0,

0, 0, 0, 0, 0, 0, 0, 0, 0, 0, 0, 0, 0,

1, 1, 0, 0, 0, 0, 0, 1, 1, 1, 0, 0, 0,

0, 0, 0, 0, 0, 0, 0, 0, 0, 0, 0, 0, 0,

0, 0, 0, 0, 0, 0, 0, 0, 0, 0, 0, 0, 0,

0, 0, 1, 1, 0, 0, 0, 0, 0, 0, 0, 0, 0,

1, 1, 0, 0, 0, 0, 0, 0, 0, 0, 1, 0, 0,

1, 0, 0, 0, 0, 0, 0, 0, 0, 0, 1, 0, 0,

0, 0, 0, 0, 0, 0, 0, 0, 0, 0, 0, 0, 0,

0, 0, 0, 0, 0, 0, 0, 0, 0, 0, 0, 0, 0,

0, 0, 0, 0, 0, 0, 0, 0, 0, 0, 0, 0, 0,

0, 0, 0, 0, 0, 0, 0, 0, 0, 0, 0, 0, 0,

0, 0, 0, 0, 0, 0, 0, 0, 0, 0, 0, 0, 0,

0, 0, 0, 0, 0, 0, 0, 0, 0, 0, 0, 0, 0,

0, 0, 0, 0, 0, 0, 0, 0, 0, 0, 0, 0, 0,

0, 0, 0, 0, 0, 0, 1, 0, 0, 0, 0, 0, 0,

0, 0, 0, 0, 0, 0, 0, 1, 1, 1, 0, 0, 1,

0, 0, 0, 0, 0, 0, 0, 0, 0, 0, 0, 0, 0,

0, 0, 0, 0, 1, 1, 1, 1, 1, 0, 0, 1, 0,

0, 0, 0, 0, 0, 0, 0, 0, 0, 0, 0, 0, 0,

0, 0, 0, 0, 0, 0, 0, 0, 0, 0, 0, 0, 0,

0, 0, 0, 0, 0, 0, 0, 0, 0, 0, 0, 0, 0,

1, 0, 0, 0, 0, 0, 0, 0, 0, 0, 0, 0, 0,

0, 0, 0, 0, 0, 0, 0, 0, 0, 0, 0, 0, 0,

0, 0, 0, 0, 0, 0, 0, 0, 0, 0, 0, 0, 0,

0, 0, 0, 0, 0, 0, 0, 0, 0, 0, 0, 0, 0,

0, 0, 0, 0, 0, 0, 0, 0, 0, 0, 0, 0, 0,

0, 0, 1, 1, 0, 0, 0, 0, 0, 0, 0, 1, 0,

0, 0, 0, 0, 0, 0, 0, 0, 0, 0, 0, 0, 0,

0, 0, 1, 0, 0, 0, 0, 0, 0, 0, 0, 0, 0,

0, 0, 0, 0, 0, 0, 0, 0, 0, 0, 0, 0, 0,

0, 0, 1, 0, 0, 0, 0, 0, 0, 0, 0, 0, 0,

0, 0, 0, 0, 0, 0, 0, 0, 0, 0, 0, 0, 0,

0, 0, 0, 0, 0, 0, 0, 0, 1, 0, 0, 0, 0,

0, 0, 0, 0, 0, 0, 0, 0, 0, 0, 0, 0, 0,

0, 0, 0, 0, 0, 0, 0, 0, 0, 0, 0, 0, 0,

0, 0, 0, 0, 0, 0, 0, 0, 0, 0, 0, 0, 0,

0, 0, 0, 0, 0, 0, 0, 0, 0, 0, 0, 0, 0,

0, 0, 0, 0, 0, 1, 0, 0, 0, 0, 1, 0, 0,

0, 0, 0, 0, 0, 0, 0, 0, 0, 0, 0, 0, 0,

0, 0, 0, 1, 0, 0, 0, 0, 0, 0, 0, 0, 0,

0, 0, 1, 1, 0, 0, 0, 1, 0, 0, 0, 1, 0,

0, 0, 0, 0, 0, 0, 0, 0, 0, 0, 0, 0, 0,

0, 0, 0, 0, 0, 0, 0, 0, 1, 0, 0, 0, 0,

0, 0, 0, 0, 1, 1, 0, 0, 1, 1, 1, 0, 0,

0, 0, 1, 1, 0, 0, 0, 0, 0, 1, 0, 0, 1,

0, 0, 0, 0, 0, 0, 0, 1, 0, 0, 0, 0, 1,

0, 0, 1, 0, 0, 0, 0, 0, 0, 0, 0, 0, 0,

0, 0, 1, 0, 0, 0, 0, 0, 0, 0, 0, 0, 0,

0, 0, 0, 0, 0, 0, 0, 0, 0, 0, 0, 0, 0,

0, 0, 0, 0, 0, 0, 0, 0, 0, 0, 0, 0, 0,

0, 0, 0, 0, 0, 0, 0, 0, 0, 0, 1, 0, 1,

0, 0, 0, 1, 0, 0, 0, 0, 0, 0, 0, 0, 0,

0, 0, 0, 0, 0, 0, 0, 0, 0, 0, 0, 0, 0,

0, 0, 0, 0, 0, 0, 0, 0, 0, 0, 1, 0, 0,

0, 0, 0, 0, 0, 0, 0, 0, 0, 0, 0, 1, 0,

0, 0, 0, 0, 0, 0, 0, 0, 0, 0, 0, 0, 0,

0, 0, 0, 0, 0, 0, 0, 1, 1, 0, 1, 0, 0,

0, 0, 0, 0, 0, 0, 0, 0, 0, 0, 0, 0, 0,

0, 0, 0, 0, 0, 0, 0, 0, 0, 0, 0, 0, 0,

0, 0, 0, 0, 0, 0, 0, 0, 0, 0, 0, 0, 0,

0, 0, 0, 0, 0, 0, 0, 0, 0, 0, 0, 0, 0,

0, 0, 0, 0, 0, 0, 0, 0, 0, 0, 0, 0, 0,

0, 0, 0, 0, 0, 0, 0, 0, 0, 0, 0, 0, 0,

0, 0, 0, 0, 0, 0, 0, 0, 0, 0, 0, 0, 0,

0, 0, 0, 0, 0, 0, 0, 0, 0, 0, 0, 0, 0,

0, 0, 0, 0, 0, 0, 0, 0, 0, 0, 0, 0, 0,

0, 0, 0, 0, 0, 0, 0, 0, 0, 1, 0, 0, 0,

0, 0, 0, 0, 1, 0, 0, 0, 0, 0, 0, 0, 0,

0, 0, 0, 0, 0, 0, 0, 0, 0, 0, 0, 0, 0,

0, 0, 0, 0, 1, 0, 0, 0, 0, 0, 0, 0, 0,

0, 0, 0, 0, 0, 0, 0, 0, 0, 0, 0, 0, 0,

0, 0, 0, 0, 0, 0, 0, 0, 0, 0, 0, 0, 0,

0, 0, 0, 0, 0, 0, 0, 0, 0, 0, 0, 0, 0,

0, 0, 0, 0, 0, 1, 0, 0, 0, 0, 0, 0, 0,

0, 0, 0, 0, 0, 1, 0, 0, 0, 0, 0, 0, 0,

0, 0, 0, 0, 1, 1, 0, 0, 0, 0, 0, 0, 0,

0, 0, 0, 0, 0, 0, 0, 0, 0, 0, 0, 0, 0,

1, 0, 1, 0, 1, 0, 0, 0, 0, 1, 1, 1, 0,

0, 0, 0, 0, 1, 0, 0, 0, 0, 0, 0, 0, 0,

0, 0, 0, 0, 0, 0, 0, 0, 0, 0, 0, 0, 0,

0, 0, 0, 0, 0, 0, 0, 0, 0, 0, 0, 1, 0,

1, 0, 0, 0, 0, 0, 0, 0, 0, 0, 0, 0, 0,

0, 0, 0, 0, 0, 0, 0, 0, 0, 0, 0, 0, 0,

0, 0, 0, 0, 0, 0, 0, 0, 0, 0, 0, 0, 0,

0, 0, 0, 0, 0, 0, 0, 0, 0, 0, 0, 0, 0,

0, 0, 1, 1, 0, 0, 0, 0, 1, 0, 0, 0, 0,

0, 0, 0, 0, 0, 0, 0, 0, 0, 0, 0, 0, 0,

0, 0, 0, 0, 0, 0, 0, 0, 0, 0, 0, 0, 1,

0, 0, 0, 0, 1, 0, 1, 0, 0, 0, 0, 0, 0,

0, 0, 0, 0, 0, 0, 0, 0, 0, 0, 0, 0, 0,

0, 0, 1, 0, 0, 0, 0, 0, 1, 0, 0, 1, 0,

0, 0, 0, 0, 0, 0, 0, 0, 0, 0, 0, 0, 0,

0, 0, 0, 0, 0, 0, 0, 0, 0, 0, 0, 0, 0,

0, 0, 0, 0, 0, 1, 1, 0, 0, 0, 0, 0, 0,

0, 0, 0, 0, 0, 0, 0, 1, 1, 0, 0, 0, 0,

0, 0, 0, 0, 1, 0, 0, 0, 0, 0, 0, 0, 0,

0, 0, 0, 0, 0, 0, 0, 0, 0, 0, 0, 0, 0,

1, 0, 0, 1, 0, 0, 0, 0, 0, 0, 0, 1, 0,

0, 1, 0, 1, 0, 1, 1, 0, 1, 0, 0, 0, 0,

0, 0, 0, 0, 0, 0, 0, 0, 0, 0, 0, 0, 0,

0, 0, 0, 0, 1, 1, 0, 0, 0, 0, 0, 0, 1,

0, 0, 0, 1, 0, 0, 0, 0, 0, 0, 1, 1, 0,

0, 0, 0, 0, 0, 0, 0, 0, 0, 1, 0, 0, 0,

0, 1, 0, 0, 0, 0, 0, 0, 0, 0, 0, 0, 0,

0, 1, 0, 0, 0, 0, 0, 0, 0, 0, 0, 0, 0,

0, 0, 0, 0, 0, 0, 0, 0, 0, 0, 0, 0, 0,

0, 0, 0, 1, 1, 0, 0, 1, 0, 0, 0, 0, 0,

0, 0, 0, 0, 0, 0, 0, 0, 0, 0, 0, 0, 1,

0, 0, 0, 1, 0, 0, 0, 0, 0, 0, 0, 0, 0,

0, 0, 0, 0, 0, 0, 1, 0, 0, 1, 0, 0, 0,

1, 1, 0, 0, 0, 0, 0, 0, 0, 0, 0, 0, 0,

1, 1, 0, 0, 0, 0, 0, 0, 0, 0, 0, 0, 1,

0, 0, 0, 0, 0, 0, 1, 0, 0, 0, 0, 0, 0,

1, 0, 0, 0, 0, 0, 0, 0, 0, 0, 0, 0, 0,

0, 0, 0, 0, 0, 0, 0, 1, 0, 0, 0, 0, 1,

0, 1, 0, 0, 0, 0, 0, 0, 0, 0, 0, 0, 0,

0, 0, 1, 0, 1, 0, 1, 0, 0, 0, 0, 1, 1,

1, 0, 0, 0, 0, 0, 1, 0, 0, 0, 0, 0, 0,

0, 0, 0, 0, 0, 0, 0, 0, 0, 0, 0, 0, 0,

0, 0, 0, 0, 0, 0, 0, 0, 0, 0, 1, 0, 0,

1, 0, 1, 0, 0, 0, 0, 0, 0, 0, 0, 0, 0,

0, 0, 0, 0, 0, 0, 0, 0, 0, 0, 0, 0, 0,

0, 0, 0, 0, 0, 0, 0, 0, 0, 0, 0, 0, 0,

0, 0, 0, 0, 1, 0, 0, 0, 0, 0, 0, 0, 0,

0, 0, 0, 0, 1, 0, 0, 0, 0, 0, 1, 0, 0,

0, 0, 0, 0, 0, 0, 0, 0, 0, 0, 0, 0, 0,

0, 0, 0, 0, 0, 0, 0, 0, 0, 0, 0, 0, 0,

0, 1, 0, 0, 0, 0, 1, 0, 1, 0, 0, 0, 0,

0, 0, 0, 1, 0, 0, 0, 0, 0, 0, 0, 0, 0,

0, 0, 0, 0, 1, 0, 0, 0, 0, 0, 1, 0, 0,

1, 0, 0, 0, 0, 0, 0, 1, 0, 0, 0, 0, 0,

0, 0, 0, 0, 0, 0, 0, 0, 0, 0, 0, 0, 0,

0, 0, 0, 0, 0, 0, 0, 1, 1, 0, 0, 0, 0,

0, 0, 0, 0, 0, 0, 0, 0, 0, 1, 1, 0, 0,

0, 0, 0, 0, 0, 0, 1, 0, 0, 0, 0, 0, 0,

0, 0, 0, 0, 0, 0, 0, 0, 0, 0, 0, 0, 1,

0, 0, 1, 0, 0, 1, 0, 0, 0, 0, 0, 0, 0,

1, 0, 0, 1, 0, 1, 0, 1, 1, 0, 1, 0, 0,

0, 0, 0, 0, 0, 0, 0, 0, 0, 0, 0, 0, 0,

0, 0, 0, 0, 1, 0, 1, 1, 0, 0, 0, 0, 1,

0, 1, 0, 0, 0, 1, 0, 0, 0, 0, 0, 0, 1,

0, 0, 0, 0, 1, 0, 0, 0, 0, 0, 0, 1, 0,

0, 0, 0, 1, 0, 0, 0, 0, 0, 0, 0, 0, 0,

0, 0, 0, 1, 0, 0, 0, 0, 0, 0, 0, 0, 0,

0, 0, 0, 0, 0, 0, 0, 0, 0, 0, 0, 0, 0,

0, 0, 0, 0, 0, 1, 1, 0, 0, 1, 0, 0, 0,

0, 0, 0, 0, 0, 0, 0, 0, 0, 0, 0, 0, 0,

1, 1, 0, 0, 0, 1, 0, 0, 0, 0, 0, 0, 0,

0, 0, 0, 0, 0, 0, 0, 0, 1, 0, 0, 1, 0,

0, 0, 1, 1, 0, 0, 0, 0, 0, 0, 0, 0, 0,

0, 0, 1, 0, 0, 0, 0, 0, 0, 0, 0, 0, 0,

0, 0, 0, 0, 0, 0, 0, 0, 1, 0, 0, 0, 0,

1, 0, 0, 1, 0, 0, 0, 0, 0, 0, 0, 0, 0,

0, 0, 0, 0, 0, 0, 0, 0, 0, 1, 0, 0, 0,

1, 0, 0, 1, 0, 0, 0, 1, 0, 1, 0, 0, 0,

0, 0, 1, 0, 1, 0, 1, 1, 1, 0, 1, 0, 0,

1, 1, 1, 0, 0, 0, 0, 0, 1, 0, 0, 0, 1,

0, 0, 0, 0, 0, 0, 0, 0, 0, 0, 0, 0, 0,

0, 0, 0, 0, 0, 0, 0, 0, 0, 0, 0, 0, 0,

1, 0, 1, 0, 1, 0, 0, 0, 0, 0, 0, 0, 0,

0, 0, 0, 0, 0, 0, 0, 0, 0, 0, 0, 0, 0,

0, 0, 0, 0, 0, 0, 0, 0, 0, 0, 0, 0, 0,

0, 0, 0, 0, 0, 0, 1, 0, 0, 1, 0, 0, 0,

0, 0, 0, 0, 0, 0, 1, 1, 0, 0, 0, 0, 1,

0, 0, 0, 0, 0, 0, 0, 0, 0, 0, 0, 0, 0,

0, 0, 0, 0, 0, 0, 1, 0, 0, 0, 0, 0, 0,

1, 0, 0, 1, 0, 0, 0, 0, 1, 0, 1, 0, 0,

0, 0, 0, 0, 0, 0, 0, 0, 0, 0, 0, 0, 0,

1, 0, 0, 0, 0, 0, 1, 1, 0, 0, 0, 0, 1,

0, 1, 1, 1, 0, 0, 0, 0, 0, 1, 0, 0, 0,

0, 0, 0, 0, 0, 0, 1, 1, 1, 1, 1, 0, 1,

0, 0, 0, 0, 1, 0, 0, 0, 0, 1, 1, 0, 0,

1, 0, 0, 0, 0, 0, 1, 1, 0, 0, 0, 1, 1,

0, 0, 0, 0, 0, 0, 0, 0, 1, 0, 0, 0, 0,

0, 0, 0, 0, 0, 1, 1, 1, 1, 1, 0, 0, 0,

0, 0, 0, 0, 1, 0, 0, 0, 0, 0, 0, 0, 1,

1, 0, 1, 0, 1, 0, 0, 1, 0, 1, 1, 0, 0,

0, 0, 0, 1, 0, 0, 0, 0, 0, 0, 0, 1, 0,

0, 1, 1, 1, 0, 0, 1, 0, 1, 1, 0, 0, 0,

0, 0, 0, 1, 0, 1, 0, 1, 0, 0, 1, 0, 1,

1, 1, 0, 1, 1, 1, 1, 0, 0, 0, 0, 0, 1,

1, 0, 0, 0, 1, 1, 0, 0, 0, 0, 0, 0, 0,

0, 0, 0, 0, 0, 0, 0, 0, 0, 0, 0, 0, 0,

0, 0, 0, 0, 0, 0, 0, 0, 0, 0, 0, 0, 0,

0, 0, 0, 0, 0, 0, 0, 1, 1, 0, 0, 1, 0,

0, 0, 1, 0, 0, 0, 1, 0, 0, 0, 0, 0, 0,

0, 0, 1, 1, 1, 0, 0, 1, 1, 0, 0, 0, 0,

0, 0, 0, 0, 0, 0, 0, 0, 0, 0, 1, 0, 0,

1, 0, 0, 0, 1, 1, 1, 0, 0, 0, 0, 0, 0,

0, 0, 0, 0, 1, 1, 0, 0, 0, 0, 0, 0, 0,

0, 0, 0, 0, 0, 0, 0, 0, 0, 0, 1, 0, 1,

1, 0, 1, 0, 0, 1, 0, 0, 0, 0, 0, 0, 1,

0, 0, 0, 0, 0, 0, 0, 0), .Dim = c(509L, 9L), .Dimnames = list(

NULL, c("UBERIS_DIPSLIDE", "STREPT_SELMA", "Uberis_Zoma",

"MISCHFLORA_DIPSLIDE", "MISCHFLORA_SELMA", "Misch_Zoma",

"NOGROWTH_DIPSLIDE", "NOGROWTH_SELMA", "NoGrowth_Zoma")))

ones <- c(1,1,1,1,1,1,1,1,1,1,1,1,1,1,1,1,1,1,1,1,1,1,1,1,1,1,1,1,1,1,1,1,1,1,1,1,1,1,1,1,1,1,1,1,1,1,1,1,1,1,1,1,1,1,1,1,

1,1,1,1,1,1,1,1,1,1,1,1,1,1,1,1,1,1,1,1,1,1,1,1,1,1,1,1,1,1,1,1,1,1,1,1,1,1,1,1,1,1,1,1,1,1,1,1,1,1,1,1,1,1,1,1,

1,1,1,1,1,1,1,1,1,1,1,1,1,1,1,1,1,1,1,1,1,1,1,1,1,1,1,1,1,1,1,1,1,1,1,1,1,1,1,1,1,1,1,1,1,1,1,1,1,1,1,1,1,1,1,1,

1,1,1,1,1,1,1,1,1,1,1,1,1,1,1,1,1,1,1,1,1,1,1,1,1,1,1,1,1,1,1,1,1,1,1,1,1,1,1,1,1,1,1,1,1,1,1,1,1,1,1,1,1,1,1,1,

1,1,1,1,1,1,1,1,1,1,1,1,1,1,1,1,1,1,1,1,1,1,1,1,1,1,1,1,1,1,1,1,1,1,1,1,1,1,1,1,1,1,1,1,1,1,1,1,1,1,1,1,1,1,1,1,

1,1,1,1,1,1,1,1,1,1,1,1,1,1,1,1,1,1,1,1,1,1,1,1,1,1,1,1,1,1,1,1,1,1,1,1,1,1,1,1,1,1,1,1,1,1,1,1,1,1,1,1,1,1,1,1,

1,1,1,1,1,1,1,1,1,1,1,1,1,1,1,1,1,1,1,1,1,1,1,1,1,1,1,1,1,1,1,1,1,1,1,1,1,1,1,1,1,1,1,1,1,1,1,1,1,1,1,1,1,1,1,1,

1,1,1,1,1,1,1,1,1,1,1,1,1,1,1,1,1,1,1,1,1,1,1,1,1,1,1,1,1,1,1,1,1,1,1,1,1,1,1,1,1,1,1,1,1,1,1,1,1,1,1,1,1,1,1,1,

1,1,1,1,1,1,1,1,1,1,1,1,1,1,1,1,1,1,1,1,1,1,1,1,1,1,1,1,1,1,1,1,1,1,1,1,1,1,1,1,1,1,1,1,1,1,1,1,1,1,1,1,1,1,1,1,

1,1,1,1,1)
