# Supplementary material for: Bayesian latent class models to determine diagnostic sensitivities and specificities of two point of care rapid tests (Selma plus, Dipslide) for the detection of Streptococcus uberis associated with mastitis in dairy cows
Source: Front Vet Sci. 2022 Dec 13;9:1062056. doi: 10.3389/fvets.2022.1062056 (PMC9792763; doi:10.3389/fvets.2022.1062056)
Supplement: Supplementary file 1 [file Data_Sheet_1.zip › S3.model.code.docx]

################################################################################################

## Model description here

################################################################################################

var p[N], q[N,8], pr[N], L[N],checks[N,16];

model {

for(i in 1:N){

q[i,1]<-pr[i]*(s1*s2*s3+covs12+covs13+covs23)+(1-pr[i])*((1-c1)*(1-c2)*(1-c3)+covc12+covc13+covc23);

q[i,2]<-pr[i]*(s1*s2*(1-s3)+covs12-covs13-covs23)+(1-pr[i])*((1-c1)*(1-c2)*c3+covc12-covc13-covc23);

q[i,3]<-pr[i]*(s1*(1-s2)*s3-covs12+covs13-covs23)+(1-pr[i])*((1-c1)*c2*(1-c3)-covc12+covc13-covc23);

q[i,4]<-pr[i]*(s1*(1-s2)*(1-s3)-covs12-covs13+covs23)+(1-pr[i])*((1-c1)*c2*c3-covc12-covc13+covc23);

q[i,5]<-pr[i]*((1-s1)*s2*s3-covs12-covs13+covs23)+(1-pr[i])*(c1*(1-c2)*(1-c3)-covc12-covc13+covc23);

q[i,6]<-pr[i]*((1-s1)*s2*(1-s3)-covs12+covs13-covs23)+(1-pr[i])*(c1*(1-c2)*c3-covc12+covc13-covc23);

q[i,7]<-pr[i]*((1-s1)*(1-s2)*s3+covs12-covs13-covs23)+(1-pr[i])*(c1*c2*(1-c3)+covc12-covc13-covc23);

q[i,8]<-pr[i]*((1-s1)*(1-s2)*(1-s3)+covs12+covs13+covs23)+(1-pr[i])*(c1*c2*c3+covc12+covc13+covc23);

######## ERROR CHECKING since (0,1) bounds could be exceeded

checks[i,1]<- s1*s2*s3+covs12+covs13+covs23;

checks[i,2]<- (1-c1)*(1-c2)*(1-c3)+covc12+covc13+covc23;

checks[i,3]<- s1*s2*(1-s3)+covs12-covs13-covs23;

checks[i,4]<- (1-c1)*(1-c2)*c3+covc12-covc13-covc23;

checks[i,5]<- s1*(1-s2)*s3-covs12+covs13-covs23;

checks[i,6]<- (1-c1)*c2*(1-c3)-covc12+covc13-covc23;

checks[i,7]<- s1*(1-s2)*(1-s3)-covs12-covs13+covs23;

checks[i,8]<- (1-c1)*c2*c3-covc12-covc13+covc23;

checks[i,9]<- (1-s1)*s2*s3-covs12-covs13+covs23;

checks[i,10]<- c1*(1-c2)*(1-c3)-covc12-covc13+covc23;

checks[i,11]<- (1-s1)*s2*(1-s3)-covs12+covs13-covs23;

checks[i,12]<- c1*(1-c2)*c3-covc12+covc13-covc23;

checks[i,13]<- (1-s1)*(1-s2)*s3+covs12-covs13-covs23;

checks[i,14]<- c1*c2*(1-c3)+covc12-covc13-covc23;

checks[i,15]<- (1-s1)*(1-s2)*(1-s3)+covs12+covs13+covs23;

checks[i,16]<- c1*c2*c3+covc12+covc13+covc23;

valid[i]<- step(1-q[i,1])*step(q[i,1])*

step(1-q[i,2])*step(q[i,2])*

step(1-q[i,3])*step(q[i,3])*

step(1-q[i,4])*step(q[i,4])*

step(1-q[i,5])*step(q[i,5])*

step(1-q[i,6])*step(q[i,6])*

step(1-q[i,7])*step(q[i,7])*

step(1-q[i,8])*step(q[i,8])*

step(1-checks[i,1])*step(checks[i,1])*

step(1-checks[i,2])*step(checks[i,2])*

step(1-checks[i,3])*step(checks[i,3])*

step(1-checks[i,4])*step(checks[i,4])*

step(1-checks[i,5])*step(checks[i,5])*

step(1-checks[i,6])*step(checks[i,6])*

step(1-checks[i,7])*step(checks[i,7])*

step(1-checks[i,8])*step(checks[i,8])*

step(1-checks[i,9])*step(checks[i,9])*

step(1-checks[i,10])*step(checks[i,10])*

step(1-checks[i,11])*step(checks[i,11])*

step(1-checks[i,12])*step(checks[i,12])*

step(1-checks[i,13])*step(checks[i,13])*

step(1-checks[i,14])*step(checks[i,14])*

step(1-checks[i,15])*step(checks[i,15])*

step(1-checks[i,16])*step(checks[i,16]);

########

L[i]<- equals(valid[i],1)*(

equals(m.uberis [i,1],1)*equals(m.uberis[i,2],1)*equals(m.uberis [i,3],1)*q[i,1]

+ equals(m.uberis [i,1],1)*equals(m.uberis[i,2],1)*equals(m.uberis [i,3],0)*q[i,2]

+ equals(m.uberis [i,1],1)*equals(m.uberis[i,2],0)*equals(m.uberis [i,3],1)*q[i,3]

+ equals(m.uberis [i,1],1)*equals(m.uberis[i,2],0)*equals(m.uberis [i,3],0)*q[i,4]

+ equals(m.uberis [i,1],0)*equals(m.uberis[i,2],1)*equals(m.uberis [i,3],1)*q[i,5]

+ equals(m.uberis [i,1],0)*equals(m.uberis[i,2],1)*equals(m.uberis [i,3],0)*q[i,6]

+ equals(m.uberis [i,1],0)*equals(m.uberis[i,2],0)*equals(m.uberis [i,3],1)*q[i,7]

+ equals(m.uberis [i,1],0)*equals(m.uberis[i,2],0)*equals(m.uberis [i,3],0)*q[i,8]

) +(1-equals(valid[i],1)) *(1e-14);

# logit(pr[i])<-intercept+slope*m.uberis[i,6];

pr[i]<-prc

## - since in a bernoulli density an observation of 1 has a likelihood of p[i]

p[i] <- L[i] / 1;## divided by a constant just to ensure all p's <1

ones[i] ~ dbern(p[i]);

}

covs12 ~ dunif(-1,1);

covs13 <- 0;

covs23 <- 0;

covc12 <- 0;

covc13 <- 0;

covc23 <- 0;

prc ~ dbeta(6.2809,13.3221); # Prev

c1 ~ dbeta(1,1); # SP Dipslide

c2 ~ dbeta(1,1); # SP Selma

c3 <- 1 # SP Zoba/Maldi

s1 ~ dbeta(1,1); # SE Dipslide

s2 ~ dbeta(1,1); # SE Selma

s3 ~ dbeta(1,1); # SE Zoba/Maldi

# intercept ~ dnorm(0,0.001);

# slope ~ dnorm(0,0.001);

logL<-sum(log(p[1:N]));

}
