# Supplementary material for: Bayesian latent class models to determine diagnostic sensitivities and specificities of two point of care rapid tests (Selma plus, Dipslide) for the detection of Streptococcus uberis associated with mastitis in dairy cows
Source: Front Vet Sci. 2022 Dec 13;9:1062056. doi: 10.3389/fvets.2022.1062056 (PMC9792763; doi:10.3389/fvets.2022.1062056)
Supplement: Supplementary file 1 [file Data_Sheet_1.zip › S4.STARD-BLCM-Checklist.docx]

|  | **Section & Topic** | **No** | **Item** | **Reported on page #** |
| --- | --- | --- | --- | --- |
|  |  |  |  |  |
|  | **TITLE OR ABSTRACT** |  |  |  |
|  |  | **1** | Identification as a study of diagnostic accuracy, using at least one measure of accuracy (such as sensitivity, specificity, predictive values, or AUC) **and Bayesian latent class models** | 1 |
|  | **ABSTRACT** |  |  |  |
|  |  | **2** | Structured summary of study design, methods, results, and conclusions  (for specific guidance, see STARD for Abstracts) | 1-2 |
|  | **INTRODUCTION** |  |  |  |
|  |  | **3** | Scientific and clinical background, including the intended use and clinical role of the **tests under evaluation** | 5-6 |
|  |  | **4** | Study objectives and hypotheses, **such as estimation of diagnostic accuracy of the tests for a defined purpose through BLCM** | 6 |
|  | **METHODS** |  |  |  |
|  | *Study design* | **5** | Whether data collection was planned before the **tests** were performed (prospective study) or after (retrospective study) | yes |
|  | *Participants* | **6** | Eligibility criteria **and description of the source population** | 7 |
|  |  | **7** | On what basis potentially eligible participants were identified  (such as symptoms, results from previous tests, inclusion in registry) | NA |
|  |  | **8** | Where and when potentially eligible participants were identified (setting, location and dates) | NA |
|  |  | **9** | Whether participants formed a consecutive, random or convenience series | convenience |
|  | *Test methods* | **10** | **Description of the tests under evaluation**, in sufficient detail to allow replication, **and/or cite references** | 8-9 |
|  |  | **11** | Rationale for choosing the **tests under evaluation in relation to their purpose** | 6 |
|  |  | **12** | Definition of and rationale for test positivity cut-offs or result categories of **the tests under evaluation**, distinguishing pre-specified from exploratory | NA |
|  |  | **13** | Whether clinical information was available to the performers or readers of **the tests under evaluation** | no |
|  | *Analysis* | **14a** | **BLCM model** for estimating measures of diagnostic accuracy | Code presented in supplementary |
|  |  | **14b** | **Definition and rationale of prior information and sensitivity analysis** | 15  Prior for prevalence based on expert knowledge and paper in preparation, sensitivity analysis for informative prevalence prior over a wide range (ie. Starting from less than 30% with mode at 10%, in consecutive steps increasing by 10% until less than 90% with a mode of 70%. For the non-informative priors for the test accuracies we also included weakly informative priors (beta(2,1) for each test |
|  |  | **15** | How indeterminate results **of the tests under evaluation** were handled | 7  Undetermined results were placed in a higher category if possible (e.g. Gram positive/Gram negative). If no classification was possible at all, results were assigned to the category “undefined”. |
|  |  | **16** | How missing data **of the tests under evaluation** were handled | NA |
|  |  | **17** | Any analyses of variability in diagnostic accuracy, distinguishing pre-specified from exploratory | We also analysed a subset of the data for which an additional culture results based on sedimentation was available |
|  |  | **18** | Intended sample size and how it was determined | Convenience |
|  | **RESULTS** |  |  |  |
|  | *Participants* | **19** | Flow of participants, using a diagram | NA, the samples come from a routine sampling, the cows are not considered as clinical patients |
|  |  | **20** | Baseline demographic and clinical characteristics of participants | NA |
|  |  | **21** | **Not applicable: the distribution of the targeted conditions is unknown, hence the use of BLCM** |  |
|  |  | **22** | Time interval and any clinical interventions between **the tests under evaluation** | NA |
|  | *Test results* | **23** | Cross tabulation of the **tests’ results (or for continuous tests results their distribution by infection stage)** | Table 1 |
|  |  | **24** | Estimates of diagnostic accuracy **under alternative prior specification** and their precision (such as 95% **credible/probability intervals**) | See sensitivity analysis |
|  |  | **25** | Any adverse events from performing **the tests under evaluation** | NA |
|  | **DISCUSSION** |  |  |  |
|  |  | **26** | Study limitations, including sources of potential bias, statistical uncertainty, and generalisability | Main limitation: all test results of Selma for Streptococcus were considered as Streptococcus uberis |
|  |  | **27** | Implications for practice, including the intended use and clinical role of **the tests under evaluation in relevant settings (clinical, research, surveillance etc.)** | Rapid point of care could be used in dairy practice |
|  | **OTHER INFORMATION** |  |  |  |
|  |  | **28** | Registration number and name of registry | NA |
|  |  | **29** | Where the full study protocol can be accessed | NA |
|  |  | **30** | Sources of funding and other support; role of funders | NA |
|  |  |  |  |  |

STARD - BLCM

STARD-BLCM stands for “Standards for the Reporting of Diagnostic accuracy studies that use Bayesian Latent Class Models” and is a modification of the STARD statement (which was recently updated to STARD2015). STARD-BLCM aims to facilitate improved quality of reporting for diagnostic accuracy studies that use Bayesian latent class models in the absence of a reference standard. The proposed modifications are relevant to both Bayesian and frequentist estimation methods but the focus is on the former.

More information for STARD (STARD2015) can be found at: [http://www.equator-network.org/reporting-guidelines/stard](http://www.equator-network.org/reporting-guidelines/stard/)

More information for STARD-BLCM can be found at: [http://www.equator-network.org/reporting-guidelines/stard-blcm](http://www.equator-network.org/reporting-guidelines/stard-blcm/)
