# Supplementary material for: Bayesian latent class models to determine diagnostic sensitivities and specificities of two point of care rapid tests (Selma plus, Dipslide) for the detection of Streptococcus uberis associated with mastitis in dairy cows
Source: Front Vet Sci. 2022 Dec 13;9:1062056. doi: 10.3389/fvets.2022.1062056 (PMC9792763; doi:10.3389/fvets.2022.1062056)
Supplement: Supplementary file 1 [file Data_Sheet_1.zip › S5.Sensitivity.analysis.pdf]

1 final model

	Lower95	Median	Upper95	SSeff	psrf
prc	0.358	0.410	0.463	30156	1
c1	0.921	0.952	0.980	28434	1
c2	0.842	0.886	0.927	30000	1
c3	1.000	1.000	1.000	NA	NA
s1	0.614	0.696	0.771	30000	1
s2	0.642	0.718	0.790	30852	1
s3	0.527	0.593	0.658	30000	1
covs12	0.053	0.072	0.090	30000	1

2 model with informative prior on prevalence, assuming a prevalence lower than 30 % with a mode at 10 %

	Lower95	Median	Upper95	SSeff	psrf
prc	0.350	0.402	0.454	29954	1
c1	0.920	0.952	0.978	29043	1
c2	0.842	0.885	0.925	30360	1
c3	1.000	1.000	1.000	NA	NA
s1	0.620	0.700	0.776	30000	1
s2	0.648	0.722	0.795	29717	1
s3	0.533	0.596	0.662	29493	1
covs12	0.052	0.071	0.089	30304	1

3 model with informative prior on prevalence, assuming a prevalence lower than 40 % with a mode at 20 %

	Lower95	Median	Upper95	SSeff	psrf
prc	0.354	0.405	0.458	31201	1
c1	0.922	0.952	0.979	30000	1
c2	0.843	0.885	0.926	30000	1
c3	1.000	1.000	1.000	NA	NA
s1	0.617	0.698	0.775	29844	1
s2	0.645	0.720	0.792	31083	1
s3	0.532	0.595	0.662	30668	1
covs12	0.052	0.071	0.090	29375	1

4 model with informative prior on prevalence, assuming a prevalence lower than 60 % with a mode at 40 %

	Lower95	Median	Upper95	SSeff	psrf
prc	0.363	0.415	0.470	31922	1
c1	0.921	0.953	0.980	30000	1
c2	0.843	0.886	0.929	30871	1
c3	1.000	1.000	1.000	NA	NA
s1	0.611	0.693	0.770	30000	1
s2	0.642	0.717	0.791	30000	1
s3	0.526	0.590	0.659	30000	1
covs12	0.054	0.072	0.091	28965	1

5 model with informative prior on prevalence, assuming a prevalence lower than 70 % with a mode at 50 %

	Lower95	Median	Upper95	SSeff	psrf
prc	0.369	0.420	0.478	31520	1
c1	0.922	0.953	0.982	30000	1
c2	0.842	0.887	0.927	30000	1
c3	1.000	1.000	1.000	NA	NA
s1	0.605	0.690	0.766	30000	1
s2	0.636	0.714	0.788	30000	1
s3	0.520	0.588	0.656	28837	1
covs12	0.054	0.072	0.091	30000	1

6 model with informative prior on prevalence, assuming a prevalence lower than 80 % with a mode at 60 %

	Lower95	Median	Upper95	SSeff	psrf
prc	0.369	0.422	0.479	30000	1
c1	0.922	0.953	0.982	30640	1
c2	0.841	0.887	0.928	30302	1
c3	1.000	1.000	1.000	NA	NA
s1	0.604	0.689	0.766	30000	1
s2	0.636	0.714	0.787	30000	1
s3	0.521	0.587	0.655	31552	1
covs12	0.053	0.072	0.091	30000	1

7 model with informative prior on prevalence, assuming a prevalence lower than 90 % with a mode at 70 %

	Lower95	Median	Upper95	SSeff	psrf
prc	0.366	0.420	0.477	29906	1
c1	0.921	0.953	0.981	30000	1
c2	0.843	0.887	0.929	29273	1
c3	1.000	1.000	1.000	NA	NA
s1	0.608	0.690	0.769	30000	1
s2	0.637	0.714	0.788	30000	1
s3	0.522	0.588	0.655	30000	1
covs12	0.054	0.072	0.091	30000	1

8 model with weakly informative prior beta(2,1) on test accuracies of Dipslide

	Lower95	Median	Upper95	SSeff	psrf
prc	0.363	0.416	0.472	29257	1
c1	0.922	0.953	0.980	30122	1
c2	0.842	0.886	0.928	30000	1
c3	1.000	1.000	1.000	NA	NA
s1	0.614	0.695	0.772	30000	1
s2	0.641	0.718	0.790	30000	1
s3	0.525	0.591	0.657	29942	1
covs12	0.053	0.072	0.090	30000	1

9 model with weakly informative prior beta(2,1) on test accuracies of Selma

	Lower95	Median	Upper95	SSeff	psrf
prc	0.362	0.416	0.471	30000	1
c1	0.921	0.953	0.980	30000	1
c2	0.843	0.887	0.928	31566	1
c3	1.000	1.000	1.000	NA	NA
s1	0.609	0.693	0.770	29869	1
s2	0.639	0.718	0.789	30000	1
s3	0.524	0.590	0.657	30000	1
covs12	0.053	0.072	0.091	30000	1

10 model with weakly informative prior beta(2,1) on sensitivity of culture

	Lower95	Median	Upper95	SSeff	psrf
prc	0.362	0.416	0.471	30993	1
c1	0.921	0.952	0.981	29449	1
c2	0.843	0.886	0.929	30105	1
c3	1.000	1.000	1.000	NA	NA
s1	0.612	0.693	0.771	31434	1
s2	0.639	0.717	0.790	30792	1
s3	0.526	0.592	0.659	29665	1
covs12	0.053	0.072	0.091	29638	1

11 model with only non-informative priors beta(1,1) for all parameters

	Lower95	Median	Upper95	SSeff	psrf
prc	0.362	0.416	0.471	30000	1
c1	0.920	0.953	0.980	30077	1
c2	0.843	0.886	0.929	30993	1
c3	1.000	1.000	1.000	NA	NA
s1	0.609	0.692	0.769	30000	1
s2	0.636	0.716	0.786	30000	1
s3	0.524	0.590	0.657	30000	1
covs12	0.053	0.072	0.091	30000	1
